# Preparation of Multifunctional Hydrogel Loaded with Isochlorogenic Acid A/Fe^3+^ Co-Assembled Nanoparticles and Its Application in Skin Wound Repair

**DOI:** 10.3390/gels12070637

**Published:** 2026-07-16

**Authors:** Hui Li, Danli Peng, Zhijia Wang, Yuping Zhang, Xingyu Yang, Yongmei Jiang, Xin Zhang, Lei Zhu, Yanlei Guo, Yongai Xiong, Gang Wang

**Affiliations:** 1 School of Pharmacy, Zunyi Medical University, Zunyi 563000, China; 18311889361@163.com (H.L.); 15120396728@163.com (D.P.); 15987478852@163.com (Z.W.); 13778362797@163.com (Y.Z.); 13765964297@163.com (X.Y.); 18385042506@163.com (Y.J.); 13708524052@163.com (X.Z.); zhuleilei@live.cn (L.Z.); 2 Chongqing Academy of Chinese Materia Medica, No.34 Nanshan Road, Nan’an District, Chongqing 400065, China; 3 Guizhou Key Laboratory of Modern Traditional Chinese Medicine Creation, Zunyi 563000, China

**Keywords:** IAA, hydrogel, metal phenolic acid nanoparticles, antioxidation, antibacterial, wound healing

## Abstract

The skin serves as the largest protective barrier organ of the human body and is easily impaired by trauma, infection and chronic diseases. Efficient wound dressings are indispensable for repairing infected wounds. Isochlorogenic acid A (IAA), the core active ingredient of Shanyinhua, has superior anti-inflammatory and antibacterial effects. However, low water solubility and weak structural stability restrict its direct application in wound treatment. In this work, IAA@Fe(III) nanoparticles (IAA@Fe(III) NPs) were synthesized through self-assembly and loaded into cross-linked amylopectin (Amy)/carboxymethyl chitosan (CMCS) (AC hydrogel) to construct Amy/CMCS@NPs composite dressings. Characterizations demonstrated that nanoparticles displayed a uniform spherical shape with a size of 114.20 ± 2.29 nm and stable coordination. The hydrogel featured a dense porous structure and outstanding mechanical performance, self-healing ability, adhesion, and swelling properties. In vitro tests proved that 50 mg/mL composite hydrogel exerted nearly 100% bacteriostatic activity against Escherichia coli (*E. coli*) and Staphylococcus aureus (*S. aureus*), with good biocompatibility, and enhanced cell migration capacity. In vivo assays indicated an 86.5% wound healing rate at day 7. This dressing could downregulate Tumor Necrosis Factor-α (TNF-α) and Interleukin-1β (IL-1β), upregulate Cluster of Differentiation 31 (CD31) and Vascular Endothelial Growth Factor (VEGF), and accelerate wound repair. This study provides a theoretical and experimental basis for the exploitation of IAA-based wound dressings and high-value utilization of Shanyinhua resources.

## 1. Introduction

Chronic infectious wounds represent a challenging and refractory disease in clinical practice. Their pathological hallmark is the persistent imbalance of the wound tissue microenvironment, characterized by protracted inflammation, tissue hypoxia, and excessive oxidative stress. This imbalance aberrantly activates inflammatory pathways and drives sustained secretion of pro-inflammatory cytokines, establishing a vicious cycle that impedes granulation tissue formation and epithelial regeneration [1]. More critically, such wounds are highly susceptible to colonization by multidrug-resistant bacteria, which secrete extracellular matrices to form biofilms that significantly enhance resistance to antibiotics and immune clearance, leading to recurrent and refractory infections [2]. Concurrently, local immune dysregulation and microcirculatory disturbances mutually reinforce each other, triggering a cascade of infection, inflammation, and tissue damage, ultimately rendering clinical treatment difficult [3,4].

Current conventional clinical approaches for chronic infectious wounds, such as debridement, dressing changes, and antibiotic therapy, all have notable limitations. Prolonged antibiotic use readily induces bacterial resistance, while single-pronged anti-inflammatory strategies fail to address both anti-infection and tissue repair needs simultaneously [5]. Traditional dressings are functionally monolithic, merely providing physical coverage and absorbing exudate, without the capacity for active and specific modulation of the pathological microenvironment [6]. An ideal wound dressing should possess excellent moisture retention, exudate absorption capacity, and good biocompatibility to foster a favorable reparative microenvironment. Hydrogels, with their three-dimensional porous networks, high water retention, and biodegradability, have become a research hotspot for advanced dressings [7]. CMCS offers biocompatibility, intrinsic antibacterial activity, and anti-inflammatory modulatory properties, yet its mechanical performance is insufficient [8]. Amy, with its highly branched structure, reinforces the gel network and enhances fluid absorption and retention [9]. Their synergistic crosslinking can construct a hydrogel carrier that combines mechanical robustness with biological functionality, providing a suitable platform for stable anchoring and sustained release of active ingredients. Although various functionalized hydrogels have been reported, such as those loaded with chlorogenic acid microspheres [10], pH/ROS-responsive nanocomposite hydrogels [11], or metal–polyphenol network hybrid hydrogels [12], the strategy of combining IAA nanoparticles with polysaccharide-based hydrogels for infected wound repair has not been documented.

IAA is a core bioactive phenolic acid derived from Shanyinhua, possessing significant antibacterial, anti-inflammatory, and antioxidant properties [13]. Its content in Shanyinhua is abundant but varies considerably among different producing regions [14]. Structurally, IAA is a dicaffeoylquinic acid (the structural formula of IAA is shown in Supplementary File Figure S1); compared with the monocaffeoylquinic acid structure of chlorogenic acid (CGA), it contains two caffeoyl groups, conferring superior antioxidant activity, such as in DPPH radical scavenging [15]. Despite its remarkable pharmacological activities, IAA’s practical biomedical application is constrained by its near insolubility in water, poor stability, and susceptibility to oxidative degradation [16]. Currently, research has achieved sustained antioxidant, anti-inflammatory, and wound-healing effects by incorporating CGA into hydrogel delivery systems via loading into ZIF-8 [10], or fabricating nanoparticles or microspheres [17]. However, studies specifically addressing IAA remain scarce. Employing nanotechnological approaches to improve the aqueous solubility and stability of IAA, while enabling its controlled delivery at wound sites, holds potential promise.

Constructing nanosystems based on metal–polyphenol coordination is an effective strategy to ameliorate the physicochemical shortcomings of polyphenolic compounds. The catechol or galloyl groups of polyphenols can coordinate with various metal ions to form stable metal–phenolic networks (MPNs) under mild and facile conditions [12]. Among the numerous metal ions, ferric ions (Fe^3+^/Fe^2+^) are ideal for constructing such nanoplatforms. Firstly, iron is an essential trace element for the human body with good biocompatibility. Secondly, the Fenton reaction mediated by iron ions can catalyze endogenous hydrogen peroxide to generate highly cytotoxic hydroxyl radicals, enabling chemodynamic sterilization [18]. More importantly, iron–polyphenol coordination has been widely utilized to construct multifunctional nanoplatforms, such as those loading curcumin or epigallocatechin gallate (EGCG), for synergistic antibacterial action and wound healing promotion [19]. Therefore, selecting ferric ions as the coordination center not only stabilizes IAA and improves its solubility but also endows the nanoparticles with additional antibacterial functions.

In this study, IAA was employed as the functional monomer to prepare nanoparticles (IAA@Fe(III) NPs) via coordination self-assembly with ferric ions. These nanoparticles were then loaded into an Amy/CMCS crosslinked hydrogel (Amy/CMCS) to construct a novel composite wound dressing. The innovations of this work are as follows: (1) proposing the use of a metal–polyphenol coordination strategy to prepare IAA-based nanoparticles, effectively addressing the bottlenecks of poor water solubility and low stability, and offering a new approach for IAA formulation development; (2) combining IAA@Fe(III) NPs with a polysaccharide hydrogel, leveraging the hydrogel’s three-dimensional porous structure, excellent moisture retention, and exudate absorption capacity to provide physical protection and a moist healing environment for the wound; (3) the composite system enables synergistic integration of IAA’s anti-inflammatory and antioxidant activities with the antibacterial effects of iron ions, effectively modulating the imbalanced wound microenvironment, disrupting bacterial biofilms, and breaking the vicious cycle of non-healing wounds; (4) opening a new avenue for the high-value utilization of active components from Shanyinhua and providing a safe and efficient novel dressing development strategy for clinical wound healing.

## 2. Results and Discussion

### 2.1. Preparation and Characterization of IAA@Fe(III) NPs

#### 2.1.1. Preparation of IAA@Fe(III) NPs

Based on metal–polyphenol coordination chemistry, this study constructed an IAA@Fe(III) NPs system. The aim was to improve the water solubility of IAA and introduce Fe(III) to enhance antibacterial efficacy. The assembly utilized the catechol groups of IAA to coordinate with Fe(III) at pH 7.0. This spontaneously formed well-structured nanoaggregates. The entire process required no organic solvents or surfactants. The preparation conditions were mild. Systematic characterization confirmed the successful construction of the nanoparticles. This provided uniform and controllable nanoscale precursors for subsequent hydrogel loading.

#### 2.1.2. Structural Characterization of IAA@Fe(III) NPs

**Figure 1:**
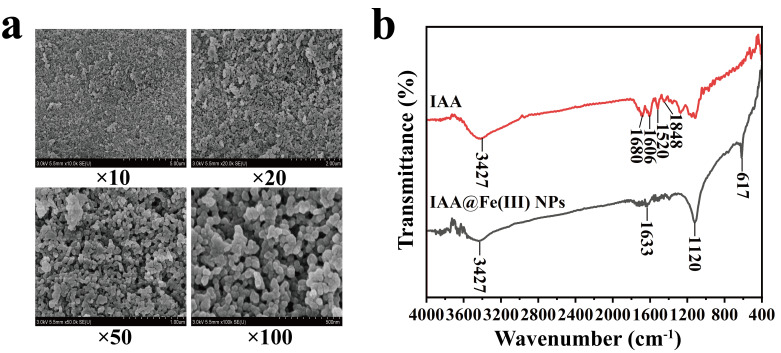
Morphology characterization and FT-IR spectra of IAA@Fe(III) NPs: (**a**) SEM image; (**b**) FT-IR spectrum.

SEM: The morphology of IAA@Fe(III) NPs was observed by SEM. As shown in Figure 1a, the particles formed by IAA and Fe^3+^ coordination were uniformly spherical with a narrow size distribution. This result was consistent with the typical spherical morphology of MPN systems reported in the literature, such as tannic acid-Fe [20]. It confirmed that IAA could also act as an effective ligand for MPN self-assembly. The uniform spherical morphology facilitated stable colloidal dispersion and prevented aggregation. It also provided a structural basis for uniform loading and controlled release when incorporated into hydrogels.

FT-IR: FT-IR was used to identify the coordination sites and bonding modes of IAA and Fe^3+^ by comparing free IAA and IAA@Fe(III) NPs (Figure 1b). The phenolic O–H peak of IAA at 3200–3550 cm^−1^ remained in the nanoparticles but with altered shape, suggesting hydroxyl participation in coordination. The carboxyl peak at 1680 cm^−1^ disappeared [21], and a new carboxylate peak appeared at 1633 cm^−1^, confirming carboxyl deprotonation and interaction with Fe^3+^. The aromatic ring peaks (1606, 1520, 1448 cm^−1^) shifted, reflecting changes in the electronic environment due to phenolic coordination. New peaks at 617 cm^−1^ (Fe–O) [22] and 1120 cm^−1^ (Fe–O–C) provided direct evidence of Fe^3+^–phenolic coordination. These features were consistent with reported gallic acid-Fe [23] and tannic acid-Fe [20] systems. This confirmed that IAA coordinated through both carboxyl and phenolic hydroxyl sites to form a metal–phenolic network, providing chemical stability for the nanoparticle structure.

**Figure 2:**
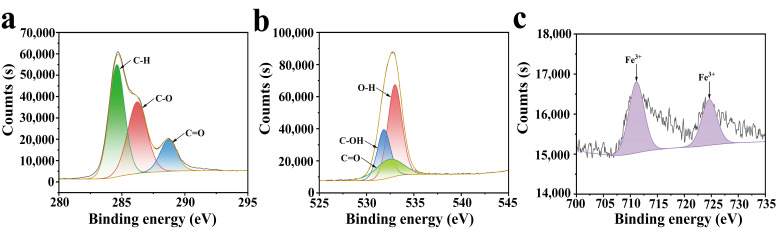
High-resolution XPS spectra of nanoparticles: (**a**) C 1s, (**b**) O 1s, and (**c**) Fe 2p.

XPS: XPS was performed to verify the coordination bonding and chemical state of Fe in IAA@Fe(III) NPs (Figure 2). The survey spectrum showed only C, O, and Fe (Figure S2), with elemental contents listed in Table 1, confirming successful Fe incorporation without impurities. The C 1s spectrum (Figure 2a) exhibited three characteristic peaks: C–H, C–O, and C=O. In the O 1s spectrum (Figure 2b), the C–OH peak shifted red to 531.84 eV, indicating charge transfer from phenolic oxygen to Fe^3+^ and formation of coordination bonds [24]. The Fe 2p spectrum (Figure 2c) showed peaks at 711.12 eV (Fe 2p_3_/_2_) and 724.61 eV (Fe 2p_1_/_2_), consistent with reported Fe^3+^–polyphenol complexes [25], confirming that Fe remained as trivalent ions without reduction. These results corroborated the FT-IR data and confirmed the IAA–Fe^3+^ coordination structure at the electronic level.

**Figure 3:**
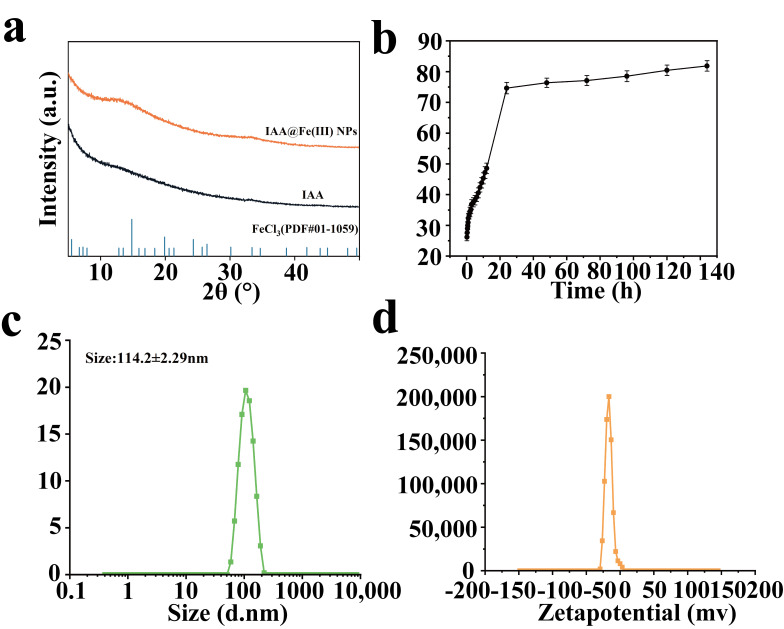
Characterizations of IAA@Fe(III) NPs: (**a**) XRD pattern; (**b**) in vitro drug release curve; (**c**) particle size distribution; (**d**) Zeta potential.

XRD: XRD was used to examine the phase composition and crystallinity of FeCl_3_, IAA, and IAA@Fe(III) NPs (Figure 3a). FeCl_3_ showed a sharp crystalline peak at 2θ = 33.407° corresponding to the (113) plane. Free IAA exhibited only a broad diffuse peak, indicating an amorphous structure. IAA@Fe(III) NPs were also amorphous, with no characteristic FeCl_3_ peaks or superposition of physical mixture patterns. This confirmed that Fe^3+^ was coordinatively embedded into the IAA network rather than simply doped. The amorphous structure favored colloidal dispersion stability and sustained drug release [26].

Particle size and Zeta potential: DLS and Zeta potential were used to evaluate the dispersion and colloidal stability of IAA@Fe(III) NPs (Figure 3c,d). DLS gave an average size of 114.20 ± 2.29 nm with a PDI of 0.064 ± 0.03, well below 0.2, confirming narrow size distribution and high uniformity, consistent with reported MPN systems (80–200 nm) [27]. The zeta potential was –16.60 ± 0.26 mV, attributed to deprotonated phenolic hydroxyl groups of IAA at neutral pH. Although this value did not reach the 30 mV threshold for high stability, combined with the PDI data, the literature reports (MPN zeta potentials of –15 to –25 mV often indicate good stability), and no observed aggregation upon standing [28], the nanoparticles were confirmed to have sufficient dispersion stability. These results provided uniform and controllable precursors for subsequent hydrogel loading.

UV: UV-Vis spectroscopy was used to verify the coordination and charge transfer between IAA and Fe^3+^ by comparing free IAA and IAA@Fe(III) NPs (Figure S3). Free IAA showed a characteristic caffeoyl π→π* absorption peak at 368 nm. This peak red-shifted after nanoparticle formation, indicating that catechol hydroxyl coordination with Fe^3+^ altered the electron density of the aromatic ring [29]. Additionally, a new absorption peak appeared at 338 nm in the nanoparticles, which was absent in free IAA. This was assigned to the ligand-to-metal charge transfer (LMCT) band [30], providing direct spectral evidence of coordination bond formation. These features were consistent with reported gallic acid-Fe^3+^ [31] and catechol-Fe^3+^ systems [32], confirming the successful construction of the IAA-Fe^3+^ coordination structure from an electronic spectral perspective.

#### 2.1.3. Performance Characterization of IAA@Fe(III) NPs

Encapsulation rate and drug loading: The encapsulation efficiency and drug loading of IAA@Fe(III) NPs were determined by ultrafiltration centrifugation. The EE was 87.29%, and the LC was as high as 93.52%, indicating high affinity between IAA and Fe^3+^ in coordination assembly. Compared with conventional polymer nanoparticles (LC < 30%) and liposomes (LC < 20%) [33], this system showed significantly higher drug loading, attributed to the self-drug-loading design where IAA itself served as the ligand framework without inert excipients. It also outperformed other MPN systems such as EGCG-Fe (34–40%) [34], owing to the high density of bis-catechol sites in IAA. These results confirmed the effectiveness of the nanodelivery system.

Drug sustained-release performance: The sustained-release performance of IAA-FeCl_3_ NPs was investigated by measuring the drug release rate at different time points, and the results are shown in Figure 3b. These nanoparticles exhibit a two-stage release kinetic characteristic: rapid drug release in the initial stage, followed by slow and continuous release. The release rate gradually levels off after 1440 min, and the cumulative release rate of IAA reaches 81.85% at 8640 min, indicating that IAA-FeCl_3_ NPs have good sustained-release potential.

### 2.2. Preparation and Characterization of Hydrogels

#### 2.2.1. Preparation of Hydrogels

Using Amy, CMCS, and borax as raw materials, AC hydrogel (Figure S4a) was prepared via a one-pot method at 65 °C. The product was moldable, appearing as a white opaque material with a fine and uniform texture, low fluidity, and high viscosity. After ultrasonic dispersion of the nanoparticles, they were used to replace deionized water, and Amy/CMCS@NPs (Figure S4b) were prepared by the same method. The obtained hydrogel was brown in color and exhibited good moldability.

#### 2.2.2. Structural Characterization of Hydrogels

**Figure 4:**
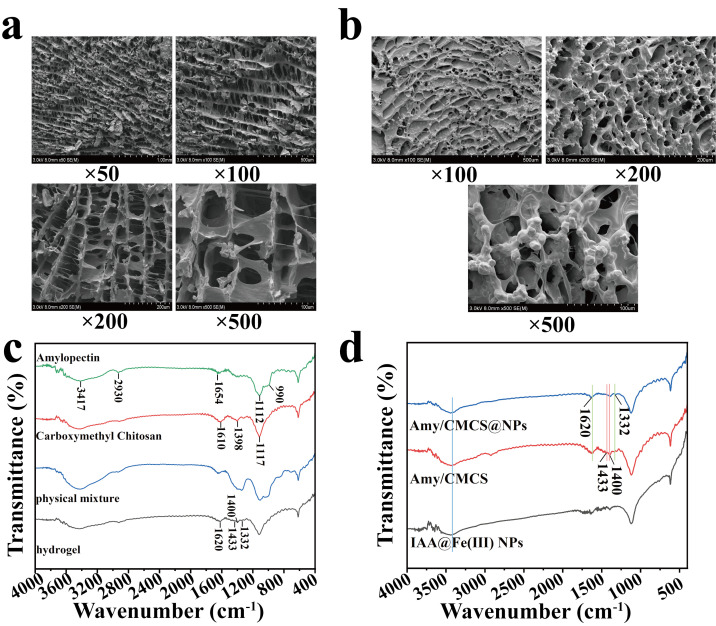
Micromorphology and FT-IR characterizations of hydrogels: (**a**) SEM images of AC hydrogel at various magnifications; (**b**) SEM images of Amy/CMCS@NPs composite hydrogel at various magnifications; (**c**) FT-IR spectrum of AC hydrogel; (**d**) FT-IR spectrum of Amy/CMCS@NPs composite hydrogel.

SEM: The microscopic porous structure of the AC hydrogel and Amy/CMCS@NPs were characterized by SEM. As shown in Figure 4a, the AC hydrogel exhibits a compact and uniform three-dimensional porous network structure with favorable air permeability and moisture retention, providing a suitable microenvironment for wound healing. As shown in Figure 4b, Amy/CMCS@NPs still maintain a dense and uniform porous structure, enabling effective absorption of wound exudate; the nanoparticles are uniformly dispersed in the gel network without obvious aggregation, confirming successful drug loading by the drug carrier. The uniform dispersion of nanoparticles strengthens the interaction between nanoparticles and gel molecules, contributing to a more stable and uniform controlled-release profile during drug release and improving the overall application performance of the gel.

FT-IR: The FT-IR analysis results (Figure 4c) show that Amy displays methine stretching vibration peaks, O–H stretching vibration peaks, O–H bending vibration peaks, C–O stretching vibration peaks, C–O–C stretching vibration peaks, and C–O–C double peaks at 2900–2950 cm^−1^, 3200–3500 cm^−1^, 1654 cm^−1^, 1030–1200 cm^−1^, 990 cm^−1^, and 1000–1180 cm^−1^, respectively [35,36]. For CMCS, overlapping O–H and N–H stretching vibration peaks are observed at 3200–3500 cm^−1^, methine stretching vibration peaks at 2900–2950 cm^−1^, C–O bond stretching vibration peaks at 1025–1152 cm^−1^, and antisymmetric and symmetric stretching vibration peaks of the COO^−^ group at 1610 cm^−1^ and 1398 cm^−1^. After the formation of AC hydrogel, asymmetric B–O–C stretching vibration peaks appear at 1332 cm^−1^ and 1433 cm^−1^, confirming the formation of a covalent cross-linking network between borax and each component [37]. Meanwhile, the stretching vibration peaks of the COO^−^ group shift to 1620 cm^−1^ and 1400 cm^−1^, which is not observed in the physical mixture. Moreover, the FT-IR results of Amy/CMCS@NPs (Figure 4d) indicate that the O–H stretching vibration peak at 3200–3500 cm^−1^ and B–O–C stretching vibration peaks at 1332 cm^−1^ and 1433 cm^−1^ are still retained, verifying the successful preparation of the hydrogel.

**Figure 5:**
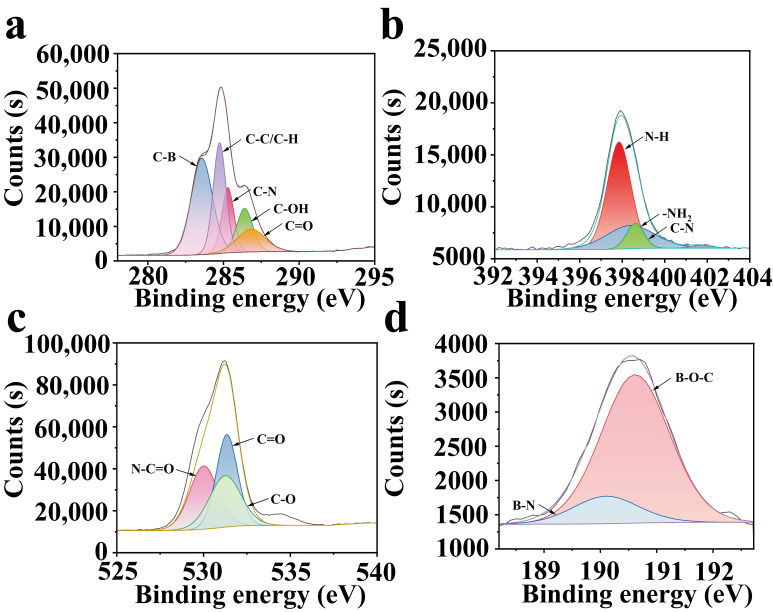
High-resolution XPS spectra of C 1s (**a**), N 1s (**b**), O 1s (**c**), and B 1s (**d**) for the AC hydrogel.

XPS: XPS was employed to characterize AC hydrogel and Amy/CMCS@NPs. This technique measures electron energy levels of various elements in the samples, allowing identification of element types, relative contents, and chemical states in the materials, providing a reliable basis for analyzing the cross-linking mechanism and nanoparticle encapsulation effect. The XPS results of AC hydrogel are displayed in Figure S5. The full spectrum clearly confirms the presence of four elements (C, N, O, and B) in the hydrogel, with characteristic peaks at C 1s (285.97 eV), O 1s (532.22 eV), N 1s (399.13 eV), and B 1s (191.84 eV). The relative contents of these four elements are listed in Table 2. To further elucidate the cross-linking process, calibration was performed using the C 1s peak at 284.8 eV as the reference binding energy. XPS peak fitting for C 1s, N 1s, O 1s, B 1s, and Fe 2p was performed using Avantage software (version 5.9931, Thermo Fisher Scientific) on data acquired with a Thermo Scientific ESCALAB 250Xi X-ray photoelectron spectrometer. The corresponding spectra are presented in Figure 5a–d. (Figure 5a–d). The C 1s spectrum exhibits five peaks assigned to C–B (283.56 eV), C–N (285.29 eV), C–OH (286.40 eV), C–C/C–H (284.74 eV), and C=O (286.83 eV) [38,39,40]. The N 1s spectrum shows four peaks corresponding to N–H (397.85 eV), C–N (398.62 eV), –NH_2_ (398.39 eV), and –CONH (401.7 eV) [41,42]. The O 1s spectrum presents three peaks attributed to C–O (531.25 eV), N–C=O (530.00 eV), and C=O (531.34 eV). The B 1s spectrum displays two peaks corresponding to B–N (190.10 eV) and B–O–C (190.60 eV) [43], verifying the successful formation of a covalent cross-linking network between borax and the hydroxyl groups of Amy and CMCS.

**Figure 6:**
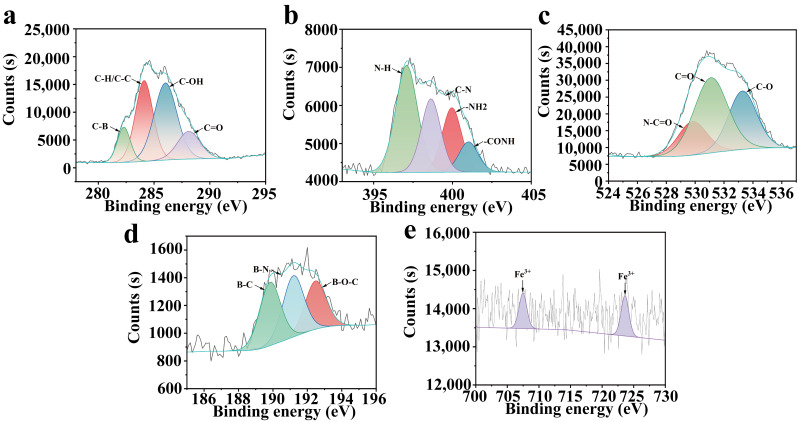
High-resolution XPS spectra of Amy/CMCS@NPs composite hydrogel, (**a**) C 1s, (**b**) N 1s, (**c**) O 1s, (**d**) B 1s, and (**e**) Fe 2p.

The XPS results of Amy/CMCS@NPs are shown in Figure S6. Compared with AC hydrogel, the full spectrum exhibits an extra characteristic peak of Fe, and a total of five elements (C, N, O, B, and Fe) are detected, with corresponding peaks at C 1s (284.83 eV), O 1s (531.76 eV), N 1s (397.93 eV), B 1s (191.90 eV), and Fe 2p (724.39 eV). The relative contents of these five elements are summarized in Table 3. Similarly, with the C 1s peak at 284.8 eV as the calibration standard, peak fitting analysis of C 1s, O 1s, N 1s, B 1s, and Fe 2p was performed using Avantage software (Figure 6a–e). The C 1s spectrum shows four peaks assigned to C–N (282.29 eV), C–C/C–H (284.11 eV), C–O (286.02 eV), and C=O (288.11 eV). The O 1s spectrum contains three peaks corresponding to N–C=O (529.81 eV), C=O (531.09 eV), and C–O (533.27 eV). The N 1s spectrum exhibits four peaks attributed to N–H (397.08 eV), C–N (399.96 eV), –NH_2_ (398.63 eV), and –CONH (401.02 eV). The B 1s spectrum presents three peaks corresponding to B–C (189.84 eV), B–O (192.47 eV), and B–N (191.19 eV). The high-resolution Fe 2p spectrum shows two characteristic peaks at 723.62 eV and 707.46 eV, confirming the successful encapsulation of nanoparticles within the hydrogel structure. Meanwhile, these results further demonstrate that borax forms a stable covalent cross-linking network with the hydroxyl groups of Amy and CMCS, and the drug-loading process does not destroy the cross-linked structure of the hydrogel.

#### 2.2.3. Performance Characterization of Amy/CMCS@NPs

Swelling is a key characteristic of hydrogel wound dressings. Both AC hydrogel and Amy/CMCS@NPs exhibit excellent swelling properties. The moist wound surface facilitates water penetration into the hydrophilic gel network; however, this process reduces the gel’s adhesion and mechanical performance [44,45]. A higher swelling rate indicates stronger water absorption capacity, enabling efficient absorption of wound exudate. Nevertheless, excessive swelling can damage the gel framework. The swelling behavior of AC hydrogel is shown in Figure S7. In the initial stage, the swelling rate is rapid, which can be attributed to two main factors: hydrogen bonding between water molecules and the gel’s hydrophilic groups (e.g., –OH), and the osmotic pressure difference between the internal gel network and the external environment. As swelling proceeds, the diffusion resistance increases and the osmotic pressure difference gradually diminishes, leading to a decelerated swelling rate until equilibrium is reached. The maximum swelling ratio of AC hydrogel was determined to be 423.13%, with no excessive swelling observed throughout the process [46].

In a pH 6.8, 0.01 M PBS solution, Amy/CMCS@NPs (Figure S8) show a rapid increase in swelling rate within 200 min and reaches equilibrium at approximately 600 min, with a maximum swelling ratio of 427.82%. After nanoparticle loading, the structure becomes more stable, allowing it to maintain balanced swelling for an extended period.

The swelling kinetics are of great significance for the development of wound dressings. The fitting results are shown in Table 4 and Table 5. Both of the hydrogels follow the second-order kinetic model (with the highest R^2^ = 0.99), indicating that the swelling rate is directly proportional to the “remaining water absorption capacity”, and the predicted values are close to the measured values. In the solution diffusion model, the AC hydrogel (Figure S9) has an *n* value of 0.54 (0.5 < *n* < 1), corresponding to non-Fickian diffusion, where the relaxation of polymer chains and the diffusion of water molecules occur simultaneously. In contrast, Amy/CMCS@NPs (Figure S10) have an *n* value of 0.043 (*n* ≤ 0.45), corresponding to Fickian diffusion. Both swelling processes are reasonable and stable, providing theoretical support for their application in wound dressings [38,39].

Viscoelasticity: Rheological test results of AC hydrogel demonstrated excellent mechanical properties and structural stability (Figure S11). Time scan analysis showed that the storage modulus G’ was consistently higher than the loss modulus G” at 25 °C, with stable values throughout the test. This indicates that the hydrogel was successfully synthesized and exhibited solid-like elastic characteristics, with sufficient internal cross-linking to enable rapid shape recovery under external force. In the shear rate test, the viscosity decreased significantly with increasing shear rate, showing typical shear-thinning behavior that endows the material with good injectability and applicability [40]. Both frequency scan and strain scan results revealed that G’ remained continuously greater than G” over the entire test range, indicating that the hydrogel’s three-dimensional network structure was stable, with excellent mechanical strength and toughness, and could maintain structural integrity without deformation or failure under dynamic stress. Overall, its rheological characteristics meet the basic mechanical requirements for wound dressings [41,42,43].

Rheological tests of Amy/CMCS@NPs (Figure S12) showed that it retained the core mechanical characteristics of the matrix hydrogel. In the time scan, G’ was always higher than G” and remained stable within 200 s, indicating that a stable three-dimensional network was formed after drug loading, with good temporal stability. In the shear rate test, the viscosity decreased significantly with increasing shear rate; the shear-thinning behavior remained unchanged, while fluidity slightly increased, which is more conducive to clinical drug administration. In the frequency scan, G’ dominated the entire frequency range, with only a slight modulus decrease in the high-frequency region. In the strain scan, G’ was greater than G” throughout the test range, and the modulus decreased under high strain, demonstrating good toughness. Compared with the AC hydrogel, the modulus of the drug-loaded hydrogel was reduced by one order of magnitude, and its stability under high frequencies and high strains was slightly weakened. However, it still maintained dominant elastic properties, and its mechanical properties could adapt to dynamic stress changes of the wound, meeting the application requirements for wound dressings.

Compression: To elucidate the reinforcement mechanism of nanoparticles on hydrogel mechanical properties, the compression behaviors of AC hydrogel and Amy/CMCS@NPs composite hydrogel were compared (Figures S13 and S14). The blank hydrogel showed a compressive strength of 40.53 kPa, fracture strain > 70%, and a Young’s modulus of only 2.78 kPa, suitable for highly dynamic wounds. After nanoparticle loading, the compressive strength surged to 468.54 kPa (approximately 11.5-fold), far exceeding the expected enhancement from simple physical filling. This reinforcement arose from chemical crosslinking reconstruction by the nanoparticles. The phenolic hydroxyl and carboxyl groups on IAA@Fe(III) NPs formed multiple hydrogen bonds and coordination bridges with polymer chains, converting the single crosslinked network into a nanoparticle–polymer hybrid network and greatly increasing crosslink density [47]. The fracture strain >80% indicated that flexibility was not sacrificed upon reinforcement, confirming the mechanism of chemical crosslinking reconstruction rather than mere physical densification.

As shown in Figure S14, the stress of Amy/CMCS@NPs increases with compressive strain, with a compressive strength of 468.54 KPa—significantly higher than that of the unloaded nanoparticle sample. This indicates that the introduction of metal-phthalic acid nanoparticles densifies the internal network cross-linking and enhances support. It is speculated that the nanoparticles form more stable chemical bonds, constructing a dense network structure. Its fracture strain exceeds 80%, meeting clinical mechanical requirements, and it exhibits higher internal network fluidity, with the stress platform located in the 60–85% strain range. The compressive Young’s modulus is 36.48 KPa, further confirming its high-density cross-linked internal network.

Thermogravimetric analysis: Thermal stability is a key factor for material application. The thermal decomposition behaviors of AC hydrogel and Amy/CMCS@NPs were analyzed using TGA, DSC, and DTG curves.

The DTG curve of AC hydrogel (Figure S15) shows that its thermal decomposition occurs in two stages, with the temperatures at which mass loss is the fastest being 137 °C and 324 °C, respectively. The TGA curve also exhibits two-stage weight loss: the mass remains essentially unchanged below 70 °C; the initial weight loss stage occurs below 260 °C, with a mass loss of approximately 12%, which is attributed to the removal of internal moisture [48]; the second weight loss stage occurs after 260 °C, involving the decomposition of side-chain structures (e.g., C=O, C-NH_2_) and the collapse of the spatial network structure [49,50]. Its DSC curve shows three endothermic peaks: the peak around 70 °C corresponds to water loss, the peak around 250 °C is the sample’s melting point (indicating good thermal stability), and the peak around 280 °C may be associated with the breakdown and rearrangement of borate ester bonds [51,52]. This hydrogel has an appropriate water content, meets the requirements for physiology and sterilization, and is suitable for wound healing.

The DTG curve of Amy/CMCS@NPs (Figure S16) shows that its thermal decomposition also occurs in two stages, with the temperatures at which mass loss is the fastest being approximately 82 °C and 320 °C. The TGA curve shows that the mass remains essentially unchanged below 60 °C, and the first weight loss stage (approximately 15%) occurs below 260 °C, attributed to water removal. The second weight loss stage occurs after 260 °C, involving chemical bond decomposition and the collapse of the spatial network structure. Its DSC curve has three endothermic peaks: the peak around 80 °C corresponds to a heat release peak caused by water loss, the peak around 252 °C is the molecular melting point, and the peak around 285 °C may be the breakdown and rearrangement of borate ester bonds. The results indicate that the thermal stability of the hydrogel is slightly improved after the introduction of nanoparticles.

#### 2.2.4. Functional Characterization of Hydrogels

Adhesion: For the application of hydrogels as wound dressings, their adhesion to biological tissues is crucial. Good adhesion ensures complete coverage of the wound area, maintains a moist environment, prevents bacterial invasion, and thereby promotes wound healing. AC hydrogel exhibits excellent adhesion performance. When applied to the finger joint, the hydrogel remains firmly adhered even when the finger is bent within the range of 30° to 90°. This is mainly attributed to the interaction between skin keratin and the hydrogel groups. Electrostatic and hydrogen bond interactions further enhance the adhesion (Figure S17a). Furthermore, this hydrogel can adhere to the surfaces of various materials such as plastic and glass and does not fall off under gravity, further confirming its adhesion ability (Figure S17b).

To quantitatively analyze the adhesion ability, a lap shear test was performed to verify its feasibility. When AC hydrogel was sandwiched between two pieces of pig skin, its adhesion strength reached 5.9 KPa, which is higher than that of commercially available dressings and suitable for skin wounds [53].

Amy/CMCS@NPs also possess excellent adhesion properties. When adhered to the surfaces of various materials, they do not fall off under gravity (Figure S18b); within the bending range of 30° to 90°, they can also firmly adhere to the finger joint (Figure S18a). The lap shear test of this hydrogel shows that its adhesion strength reaches 29.6 KPa, which is not only higher than that of commercially available dressings but also superior to that of the hydrogel without the addition of IAA-FeCl_3_ NPs. Research indicates that the addition of the metal–phenolic acid complex system can enhance the cohesion of the hydrogel. The excellent adhesion characteristics of both hydrogels endow them with potential application value in the field of wound healing.

Self-healing: Both the AC hydrogel and Amy/CMCS@NPs exhibit excellent self-healing capabilities. When an intact AC hydrogel sample is divided into two parts and brought into contact, a complete hydrogel can be reformed after a period of time (Figure S19); similarly, when Amy/CMCS@NPs are cut into two parts and brought into contact, they can also re-polymerize into a complete gel after a period of time (Figure S20).

Drug release: To investigate the drug release behavior, the dialysis method was used to test the drug release. As shown in Figure S21, the drug release increases with the extension of time. At 720 min, the cumulative drug release reaches 61.39%, and after 2160 min, the drug release tends to level off, with a release rate exceeding 80%.

### 2.3. Study on Antibacterial Properties and Promotion of Wound Healing in Mice by Hydrogel

#### 2.3.1. The Antibacterial Properties of Hydrogels

**Figure 7:**
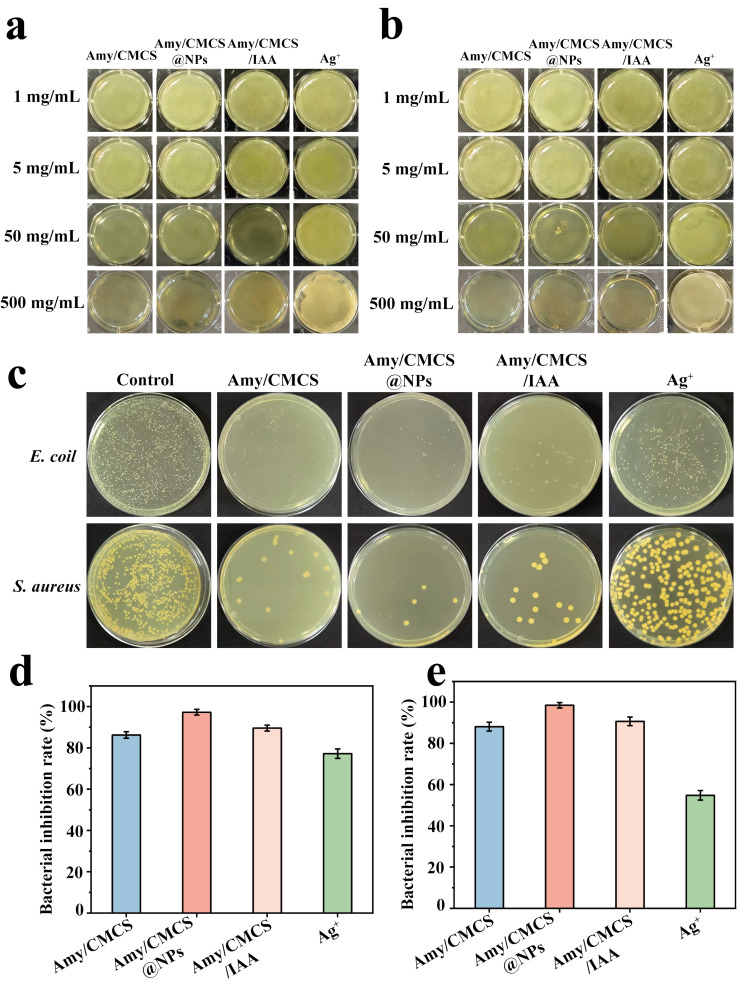
In vitro antibacterial activity evaluation of Amy/CMCS blank hydrogel and Amy/CMCS@NPs composite hydrogel: (**a**) MIC determination against *E. coli*; (**b**) MIC determination against *S. aureus*; (**c**) plate antibacterial effect of different treatment groups against two bacteria; (**d**) antibacterial rate against *E. coli*; (**e**) antibacterial rate against *S. aureus.*

The minimum inhibitory concentration (MIC) refers to the lowest concentration of an antibacterial agent that can inhibit the visible growth of target bacteria under standard conditions, and it is an important indicator for evaluating the antibacterial effect of antibacterial materials [54]. In this experiment, the hydrogel supplemented with IAA-FeCl_3_ NPs was used as the research object, with a concentration gradient of 1, 5, 50, and 500 mg/mL set to investigate its antibacterial effect on *E. coli* (Figure 7a) and *S. aureus* (Figure 7b). After 24 h of cultivation, the results demonstrated that the hydrogel at a concentration of 50 mg/mL could completely inhibit the growth of both strains, with no obvious colonies observed on the plates. On this basis, the plate counting method was employed for quantitative analysis of the antibacterial effect (Figure 7c). Compared with the blank hydrogel, the hydrogel loaded with IAA-FeCl_3_ NPs exhibited significantly enhanced antibacterial performance, and the antibacterial rates against *E. coli* and *S. aureus* were both close to 100% (Figure 7d,e), indicating that the introduction of these nanoparticles can endow the hydrogel with excellent antibacterial activity.

#### 2.3.2. Evaluation of the Biocompatibility of Hydrogels

**Figure 8:**
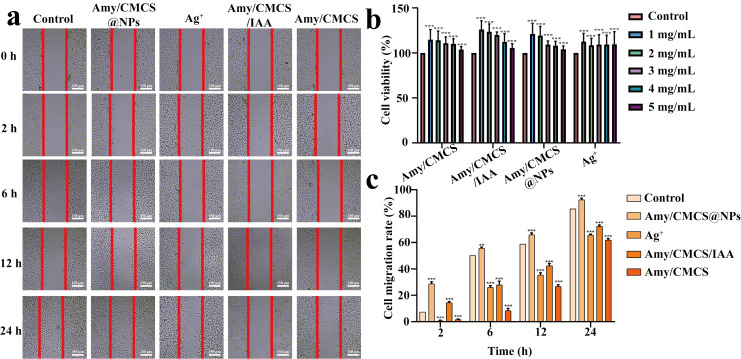
In vitro cytocompatibility and pro-migration ability evaluation of Amy/CMCS@NPs composite hydrogel: (**a**) representative images of scratch assay in different treatment groups (0–24 h); (**b**) L929 cell viability (CCK-8 assay); (**c**) quantitative analysis of cell migration rate ( ** *p* < 0.01, *** *p* < 0.001 vs. Control group).

Biocompatibility is a primary prerequisite for hydrogels as wound dressings. Cytotoxicity of the hydrogels against L929 fibroblasts was evaluated by Thiazolyl blue (MTT) assay (Figure 8b). After 48 h treatment with hydrogel extracts at various concentrations, cell viability in all groups exceeded 100%, indicating no cytotoxicity and a certain pro-proliferative tendency. The positive control Ag^+^ group also showed >100% viability, consistent with reported good tolerance of mammalian cells at the tested concentration.

Cell migration is critical for wound re-epithelialization. Scratch assays (Figure 8a,c) showed that all hydrogel materials promoted directional cell migration to varying degrees compared with the blank group. Migration was not obvious within the first 2 h. After 6 h, scratch closure began in the experimental groups. By 24 h, cells in the nanoparticle hydrogel (Amy/CMCS@NPs) group nearly completely covered the scratch area, with a migration rate significantly superior to that of the blank hydrogel and Ag^+^ groups. This result was consistent with the MTT findings and functionally confirmed good biocompatibility and pro-healing potential of the hydrogels [55].

Regarding the pro-migration mechanism, it may involve sustained release of active components. Oligosaccharides released from CMCS degradation can act as cell signaling molecules to activate integrin-mediated cell adhesion and migration. Fe^3+^ may promote fibroblast proliferation and migration through the PI3K/Akt pathway [56]. Additionally, the anti-inflammatory activity of IAA can suppress excessive inflammation, indirectly creating a favorable microenvironment for cell migration. These synergistic mechanisms will be further validated in subsequent studies.

#### 2.3.3. Evaluation of the Area of Wound Repair in Mice Treated with Hydrogel

**Figure 9:**
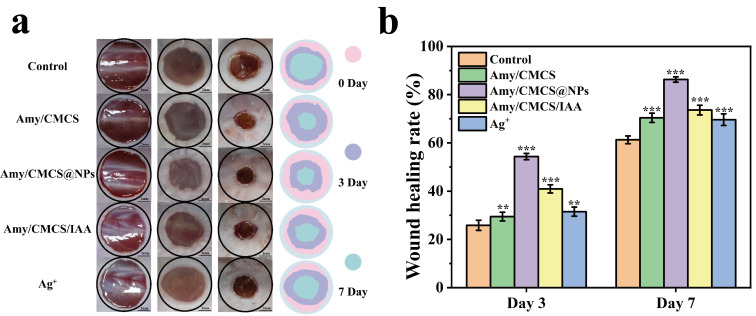
In vivo evaluation of Amy/CMCS@NPs composite hydrogel on wound healing in ICR mice: (**a**) representative images of wounds in different treatment groups on days 0, 3, and 7; (**b**) statistical histogram of wound healing rate at each time point (** *p* < 0.01, *** *p* < 0.001 vs. Control group).

In this study, a dorsal skin defect model with an 8-mm-diameter wound was constructed in male ICR mice. Five experimental groups were designed, including the untreated model group, drug-free Amy/CMCS group, IAA-FeCl_3_ NPs-loaded Amy/CMCS@NPs group, IAA-supplemented Amy/CMCS/IAA group, and Ag^+^ gel group. Wound conditions were photographed and recorded on days 0, 3, and 7 post-modeling, with relevant results presented in Figure 9a. On day 3 of treatment, the wound healing rates of the five groups were 29.5%, 31.5%, 54.8%, 40.9%, and 40.9%, respectively. All treatment groups entered the inflammatory stage with neovascularization observed. On day 7, the Amy/CMCS@NPs group achieved a high healing rate of 86.5% (Figure 9b), which was markedly superior to that of other groups, verifying its prominent efficacy in accelerating wound repair.

#### 2.3.4. Histological Evaluation by Hematoxylin–Eosin (H and E) Staining and Masson Staining

Wound healing is a dynamic and continuous physiological process. In this study, H and E staining was used to observe tissue morphology and evaluate the promoting effect of different hydrogels on wound healing. Meanwhile, Masson staining was employed to detect collagen deposition at the wound site and explore its impact on tissue repair.

**Figure 10:**
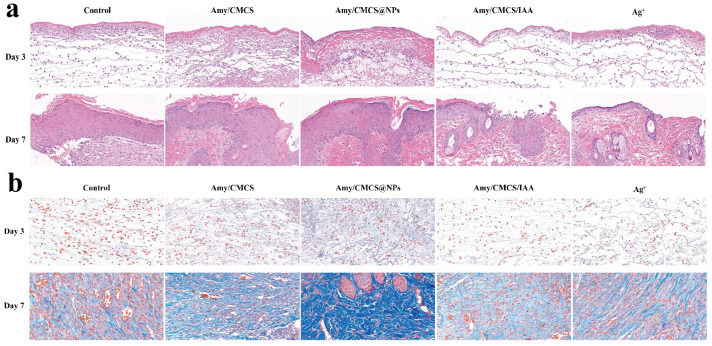
Histological evaluation of wound tissue repair in ICR mice treated with Amy/CMCS@NPs composite hydrogel: (**a**) H&E staining and (**b**) Masson staining analysis on days 3 and 7. H&E staining: nuclei appear purple, cytoplasm and extracellular matrix appear pink; Masson staining: collagen fibers appear blue, muscle fibers and red blood cells appear red.

The H and E staining results are shown in Figure 10a. Wound tissues from each experimental group were stained and analyzed on the third and seventh days after modeling. On the third day, all experimental groups exhibited obvious tissue defects, with loose subcutaneous tissue, discontinuous tissue structure [57], and an initially formed dermis layer while the epidermis layer had not yet fully developed. Each group showed only a small amount of granulation tissue formation, accompanied by inflammatory cell infiltration [58]. On the seventh day, re-epithelialization occurred in all groups, forming a relatively complete epidermis layer. A large amount of granulation tissue appeared in the wounds, with new capillaries, hair follicles, and glands visible, indicating that the wounds were in the re-epithelialization and tissue remodeling stage.

As the main component of connective tissue, collagen is of great significance for granulation tissue formation, new blood vessel generation, tissue remodeling, and enhancing the tensile strength of new tissues [59]. The Masson staining results are shown in Figure 10b. On the third day, red dot-like areas (red blood cells and inflammatory cells) were observed on the wound surfaces of all groups, and a small amount of collagen deposition was detected in each group. The IAA@Fe(III) NPs group showed more collagen deposition than the other experimental groups. On the seventh day, collagen deposition in all experimental groups increased significantly; compared with other experimental groups, the IAA-FeCl_3_ NPs group had more, denser, and more organized collagen deposition at the wound site, further confirming that this group of hydrogels can effectively promote collagen synthesis and accelerate wound tissue repair.

#### 2.3.5. Immunohistochemical Histological Evaluation

The inflammatory response is a key factor influencing the wound repair process. IL-1β and TNF-α, as core pro-inflammatory factors in the inflammatory response, play significant roles in the inflammatory process [60,61]. Among them, IL-1β can promote leukocyte activation, induce the release of inflammatory mediators, and enhance vascular permeability; its excessive expression will exacerbate the inflammatory response and aggravate tissue damage and pain. TNF-α is secreted by macrophages and participates in the body’s inflammatory response and immune regulation. In this study, the inflammatory status of wounds in different treatment groups was analyzed through immunohistochemical staining of inflammatory factors, and the regulatory effects of each treatment regimen on the inflammatory process were systematically evaluated.

**Figure 11:**
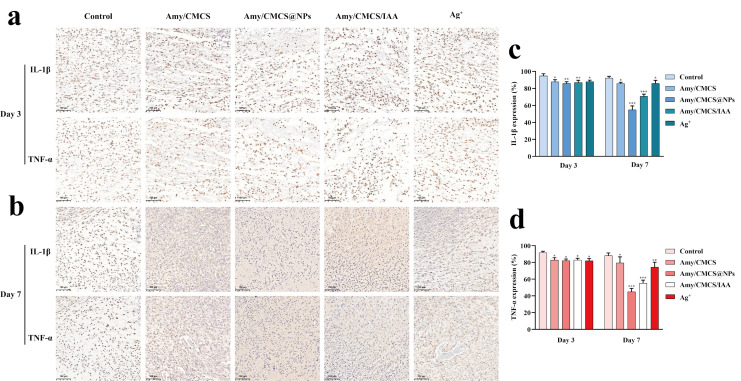
Expression analysis of pro-inflammatory cytokines (IL-1β and TNF-α) in wound tissues: (**a**) Day 3 and (**b**) Day 7 immunohistochemical staining; (**c**) quantitative analysis of relative IL-1β expression; (**d**) quantitative analysis of relative TNF-α expression. Immunohistochemical staining: positive expression of IL-1β and TNF-α appears brown, while cell nuclei are counterstained blue. Scale bar = 100 μm. (Note: *** *p* < 0.001, ** *p* < 0.01, * *p* < 0.05 vs. Control).

The experimental results are shown in Figure 11a,c. On the third day, a large amount of IL-1β and TNF-α was expressed in the wounds of all groups, indicating that all experimental groups were in the early inflammatory stage of wound repair at this time. However, it can be seen that the expression levels of the two pro-inflammatory factors in the Amy/CMCS@NPs treatment group were significantly lower than those in other experimental groups, indicating that under the intervention of IAA-FeCl_3_ NPs, this group could inhibit the expression of inflammatory factors in the early healing stage and enter the transition period of wound healing earlier.

The results on the seventh day are shown in Figure 11b,d. The expression levels of IL-1β and TNF-α in the Amy/CMCS@NPs group were extremely low, while the other treatment groups still showed relatively weak expression but at higher levels than this group. This confirms that the AC hydrogel loaded with IAA-FeCl_3_ NPs performed better in promoting the transition of wounds from the inflammatory stage to the remodeling stage. Its anti-inflammatory advantage stems from the composition of the hydrogel: CMCS itself has certain anti-inflammatory and bactericidal effects and can inhibit the expression of IL-1β and TNF-α; meanwhile, IAA, an active component in the nanoparticles loaded in the hydrogel, has antioxidant effects and can interact with wound cells to further inhibit the secretion of inflammatory factors.

#### 2.3.6. Immunofluorescence

**Figure 12:**
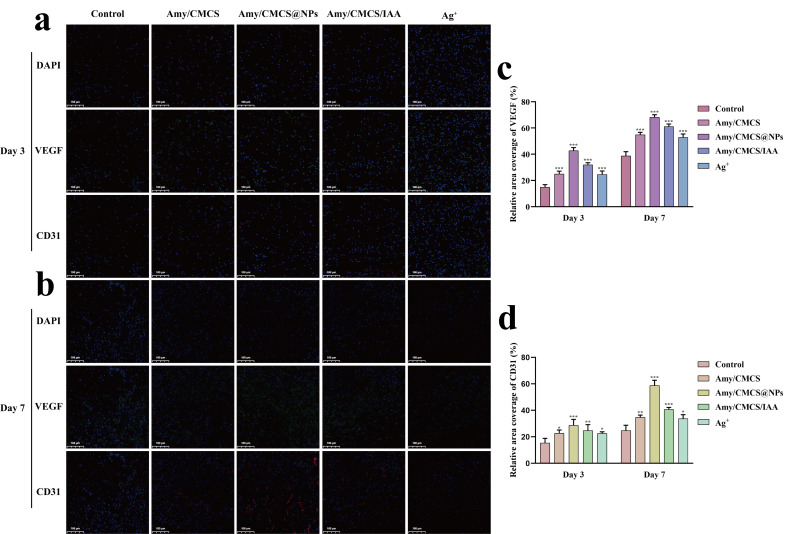
Immunofluorescence staining of CD31 and VEGF in wound tissues: (**a**) Day 3 and (**b**) Day 7; (**c**) quantification of relative fluorescence intensity of VEGF; (**d**) quantification of relative fluorescence intensity of CD31. Immunofluorescence staining: VEGF appears green, CD31 appears red, and cell nuclei are counterstained blue (DAPI). Scale bar = 100 μm. (Note: *** *p* < 0.001, ** *p* < 0.01, * *p* < 0.05 vs. Control).

CD31 is a transmembrane protein expressed in endothelial cells, and VEGF is vascular endothelial growth factor. In this study, dual immunofluorescence staining of CD31 and VEGF in wound tissues was used to evaluate the formation of new blood vessels and assess the hydrogel’s effect on promoting angiogenesis. As shown in Figure 12, on the third day, the expression of CD31 and VEGF was very weak in all experimental groups; however, compared with other experimental groups, the fluorescence expression in the IAA-FeCl_3_ NPs group was slightly stronger. On the seventh day, the expression of CD31 and VEGF increased in all experimental groups. Similarly, the fluorescence expression in the IAA-FeCl_3_ NPs group was stronger than that in other groups. These results indicate that Amy/CMCS@NPs are conducive to promoting the formation of new blood vessels at the wound site, thereby facilitating wound healing.

### 2.4. Antioxidant Properties and Mechanisms of IAA

The antioxidant activity of IAA has been well documented in the literature. For direct radical scavenging, IAA at 10 μg/mL showed 97.04% DPPH radical scavenging and 60.01% hydroxyl radical scavenging, with reducing power superior to vitamin C, confirming the efficient hydrogen donation and electron transfer capacity of its catechol structure [62]. In cellular models, IAA significantly reduced ROS and MDA levels and increased SOD activity in oleic-acid-induced HepG2 cells, effectively alleviating oxidative stress [63]. At the molecular level, Li et al. (2026) [64] used C. elegans models and found that IAA promoted SKN-1 (Nrf2 homolog) nuclear translocation (23.86% → 43.92%) and upregulated downstream antioxidant genes gst-4 and gcs-1, with this effect completely abolished in skn-1-deficient mutants. Molecular docking further revealed high-affinity binding of IAA to the Keap1 active pocket (binding energy –7.807), occupying the Nrf2 binding site through hydrogen bonds, π–π stacking, and electrostatic interactions, competitively releasing Nrf2 to initiate antioxidant enzyme transcription. This mechanism was also validated in MRC-5 cells. Based on these mechanisms, in the IAA@Fe(III) NPs hydrogel designed in this study, IAA directly scavenges wound ROS via phenolic hydrogen donation, while the Fe^3+^ coordination network stabilizes iron ions and suppresses Fenton reactions, synergistically alleviating oxidative stress. This provides theoretical support for the hydrogel to promote wound healing through an “antioxidant–anti-inflammatory” synergistic pathway.

### 2.5. The Degradation Behavior of Hydrogels and Its Significance in the Application of Wound Dressings

The crosslinking network of AC hydrogel is based on boronate ester dynamic covalent bonds, which are responsive to pH and ROS. In the wound microenvironment (weakly acidic, high ROS), these bonds undergo reversible cleavage, leading to gradual hydrogel degradation. Studies have shown that boronate ester hydrogels degrade faster at acidic pH (5.5) than at neutral pH, and they also accelerate in H_2_O_2_-containing media, confirming their stimuli-responsive degradation behavior [65]. AC hydrogel is expected to follow this pattern, enabling autonomous accelerated degradation and on-demand drug release in the wound microenvironment. Moderate degradation is important for dressing applications. First, degradation accompanies the gradual release of IAA@Fe(III) NPs, achieving sustained delivery and avoiding burst release [66]. Second, when degradation matches tissue regeneration, it provides transitional support for new tissue—too fast exposes the wound, while too slow hinders tissue ingrowth [67]. Third, CMCS and Amy are natural polysaccharides, and their degradation products are nontoxic oligosaccharides that can be metabolically cleared, offering better safety than synthetic polymers. However, the hydrolysis sensitivity of boronate esters poses challenges for storage stability, and in vitro conditions cannot fully simulate the complex in vivo environment. The long-term safety of phenylboronic acid derivatives also requires systematic evaluation. Future work should include long-term in vivo degradation tracking and large-animal studies to support clinical translation [68].

### 2.6. Evaluation of the Skin Irritation of the Formulation

Skin irritation is an essential safety evaluation for clinical translation of wound dressings. In a full-thickness skin defect model in ICR mice, skin conditions around the wound and under the dressing were observed during daily dressing changes. No obvious erythema, edema, desquamation, or behavioral abnormalities were seen in any Amy/CMCS@NPs hydrogel-treated group over the 14-day observation period, preliminarily indicating good skin tolerance. However, systematic skin irritation evaluation—such as intradermal reaction tests or acute contact dermatitis tests per ISO 10993-10 [69]—was not conducted in this study. This was mainly because the experimental design focused on wound healing efficacy and preliminary biocompatibility, without separate irritation assessment independent of the wound model. Moreover, the inflammatory microenvironment of the wound bed itself may interfere with or mask irritation responses, so standardized testing on normal skin remains indispensable. The literature shows that various hydrogels have demonstrated good safety in formal evaluations. Zakzak et al. (2024) [70] confirmed carbomer-based hydrogels as non-irritating through in vitro cytotoxicity, HET-CAM, and nude mouse skin tests. Smeu et al. (2025) [71] confirmed betulinic acid hydrogels as non-irritating using 3D EpiDerm microtissues and HET-CAM. Martins et al. (2025) [72] developed a curcumin/polyhexamethylene biguanide nanoemulsion hydrogel that was also non-irritating by HET-CAM. Lapmanee et al. (2025) [73] performed rabbit skin irritation tests on gelatin-based composite hydrogels, reporting a primary irritation index of 0.62, within the slightly irritating range. These studies suggest that skin irritation of hydrogel dressings is generally well controllable. In summary, although this study preliminarily observed no obvious irritation from Amy/CMCS@NPs hydrogel, standardized evaluation still needs completion. Subsequent rabbit skin irritation tests and guinea pig sensitization tests per ISO 10993 series will be performed to comprehensively assess skin safety and provide more robust data for clinical translation.

### 2.7. The Potential Molecular Mechanism of Amy/CMCS@NPs Hydrogel in Down-Regulating TNF-α and IL-1β

This study found that Amy/CMCS@NPs hydrogel significantly downregulated the pro-inflammatory cytokines TNF-α and IL-1β in wound tissue, but the molecular mechanism remains unclear. Based on literature evidence and the characteristics of our material system, we propose the following multi-pathway synergistic mechanistic hypothesis: (1) IAA-mediated NF-κB pathway inhibition. IAA, as a dicaffeoylquinic acid compound structurally similar to chlorogenic acid, has been reported to inhibit IκBα phosphorylation and degradation, blocking NF-κB nuclear translocation and thereby downregulating transcription of TNF-α, IL-1β, and IL-6 [74]. This pathway is the primary molecular basis for the anti-inflammatory activity of IAA. (2) Fe^3+^-mediated ROS scavenging and indirect anti-inflammation. Although Fe^3+^ itself is not a direct antioxidant, the metal–polyphenol coordination network sequesters free Fe^3+^ in the coordination structure, reducing the capacity of free iron ions to catalyze Fenton reactions and generate hydroxyl radicals, thus lowering ROS levels [75]. Since ROS are upstream signals for NF-κB activation, reduced ROS indirectly inhibits NF-κB pathway activation. (3) Synergistic bioactivity of CMCS. CMCS has been reported to moderately regulate macrophage polarization through interaction with Toll-like receptor 4 (TLR4), promoting M1-to-M2 transition and downregulating inflammatory cytokine expression [76]. These three components act synergistically to regulate pro-inflammatory cytokines. Additionally, the three-dimensional porous structure of the hydrogel may physically reduce mechanical stimulation and bacterial contact with the wound, indirectly lowering local inflammation. This hypothesis systematically links material chemical properties with biological effects. However, direct molecular evidence still requires verification of key NF-κB pathway proteins (p-IκBα, p-p65) and macrophage polarization markers (iNOS, CD206) by Western blot or immunofluorescence staining.

### 2.8. Comparison with Previous Research Work and Its Limitations

This study successfully constructed a composite hydrogel (NPs/AC hydrogel) based on IAA-Fe^3+^ metal-phenolic network nanoparticles (IAA@Fe(III) NPs) and systematically validated its potential for wound healing. This design aligns with recent strategies using MPNs for multifunctional wound repair materials. Gong et al. (2025) used EGCG-Fe^3+^ nanoparticles to load multiple active molecules for diabetic foot ulcer treatment [19]. Zhang et al. (2024) [77] coated tannic acid-Fe^3+^ networks onto copper sulfide nanozymes for antioxidant and photothermal antibacterial properties. Hu et al. [78] (2026) constructed curcumin-loaded copper-iron bimetallic nanoparticle hydrogels for chronic wound microenvironment reprogramming. These studies collectively demonstrate the great potential of MPNs in wound repair. Compared with these works, our study has unique advantages. First, using IAA from Shanyinhua as the MPN ligand expands the ligand source and utilizes the anti-inflammatory and antibacterial activities of IAA for synergistic therapy. Second, the dynamic covalent hydrogel network composed of Amy and CMCS endows the material with excellent self-healing, adhesion, and mechanical properties, providing an ideal three-dimensional environment for cell behavior. Mechanistically, our results are comparable with literature reports. MPNs scavenge ROS to break the inflammatory vicious cycle, similarly seen in Gong et al. (2025) [19] and Hu et al. (2026) [78]. The sustained release of metal ions together with IAA upregulating VEGF and CD31 to promote angiogenesis also echoes findings by Zhang et al. (2024) [77] and Hu et al. (2026) [78], suggesting that NPs/AC hydrogel accelerates wound healing through multiple synergistic pathways including antibacterial, anti-inflammatory, and pro-angiogenic effects. This study also has limitations. The deep molecular pathways by which IAA@Fe(III) NPs regulate macrophage polarization and angiogenesis (e.g., NF-κB, PI3K/AKT) have not been systematically elucidated. In vivo experiments were only performed in healthy mouse acute wound models, without involving more clinically relevant diabetic or infected chronic wound models. Long-term in vivo distribution, metabolism, and toxicity of the nanoparticles are insufficiently evaluated. The coupling between precise release kinetics of active components and material degradation also requires further study. In summary, this study provides new ideas for the deep development of Shanyinhua and an experimental basis for designing multifunctional wound repair materials. Future work will focus on chronic wound model validation and molecular mechanism investigation to advance clinical translation.

## 3. Conclusions

This study utilized CMCS and Amy as raw materials to construct an AC hydrogel via borate ester bond cross-linking. This hydrogel features a dense three-dimensional network structure and exhibits excellent self-healing, adhesion, mechanical properties, and swelling capacity, laying a foundation for the development of wound dressings. Meanwhile, IAA@Fe(III) NPs with good dispersibility were synthesized through the coordination interaction between IAA and Fe^3+^, which possess both drug sustained-release and free radical scavenging capacities. Combining these two components yielded the Amy/CMCS@NPs hydrogel, where the nanoparticles were uniformly dispersed within the gel network, with a cumulative drug release rate exceeding 80% within 36 h. In vitro experiments demonstrated its excellent antibacterial activity and good biocompatibility, while in vivo experiments confirmed that this hydrogel can accelerate skin wound healing by regulating inflammatory responses, promoting angiogenesis, and enhancing collagen deposition. In conclusion, the Amy/CMCS@NPs hydrogel is a highly promising wound dressing; however, further research on complex pathological wound models is required to verify its broad applicability and improve its translational potential.

## 4. Materials and Methods

### 4.1. Materials

The main reagents used in this experiment were all of analytical grade. CMCS (CAS 83512-85-0, degree of substitution ≥80%), Amy (CAS 9037-22-3), and FeCl3·6H_2_O (CAS 10025-77-1, 99.9%) were purchased from Shanghai Macklin Biochemical Co., Ltd. (Shanghai, China). Borax (CAS 1303-96-4, 99.9% metals basis) was purchased from Shanghai Aladdin Biochemical Technology Co., Ltd. (Shanghai, China). IAA (CAS 2450-53-5, HPLC > 98%) was purchased from Chengdu Ruifenshi Biotechnology Co., Ltd. (Chengdu, China).

Experimental cells and biological reagents: L929 cells (BC-C-MI-026) were purchased from Nanjing Shenghang Biotechnology Co., Ltd. *E. coli* (BNCC336902) and *S. aureus* (BNCC381462) were purchased from Beina Biotechnology. MTT (CAS 298-93-1) and penicillin–streptomycin double antibody (100 U/L, P1400) were purchased from Beijing Solarbio Technology Co., Ltd. Mouse IL-1β, TNF-α, CD31, and VEGF detection kits were all purchased from Wuhan Servicebio Technology Co., Ltd. The 4% paraformaldehyde (BL539A) and PBS (BL601A) were purchased from Beijing Lanjieke Technology Co., Ltd.

SPF-grade 6-week-old male ICR mice (body weight 30 g) were purchased from Chongqing Tengxin Biotechnology Co., Ltd. The mice were housed at 22 ± 2 °C and relative humidity 55 ± 10%, with free access to food and water. All breeding materials, feed, and drinking water met experimental standards. All animal experiments strictly followed the Guide for the Care and Use of Laboratory Animals of Zunyi Medical University. The experimental protocol was approved by the university’s Animal Ethics Committee (approval number: ZMUAEC-2603-641). The 3R principle was followed throughout the experiment to minimize animal suffering and reduce the number of animals used. All surgical procedures were performed under sterile conditions. After the experiment, the mice were euthanized by cervical dislocation.

### 4.2. Preparation and Characterization of IAA@Fe(III) NPs

#### 4.2.1. Preparation of IAA@Fe(III) NPs

Using IAA and FeCl_3_·6H_2_O as raw materials, IAA@Fe(III) NPs were prepared. Specifically, 15 mg of IAA was accurately weighed and ultrasonically dispersed in 5 mL of deionized water. The pH was then adjusted dropwise to 7.0 with 0.1 M NaOH to completely dissolve the IAA until a clear and transparent solution was obtained, yielding the IAA solution. Separately, 30 mg of FeCl_3_·6H_2_O was dissolved in 15 mL of deionized water to prepare the iron salt solution (the mass ratio of IAA to FeCl_3_·6H_2_O was 1:2). The iron salt solution was placed in a 60 °C water bath with magnetic stirring at 300 rpm, and the IAA solution was slowly added dropwise at a rate of approximately 1 mL/min. After the addition was completed, the reaction was allowed to continue with stirring for 6 h. Upon completion of the reaction, the product was allowed to stand at 4 °C overnight, then centrifuged at 4500 rpm for 20 min. The precipitate was washed three times with deionized water and subsequently freeze-dried to obtain the lyophilized powder of IAA@Fe(III) NPs [79].

#### 4.2.2. Structural Characterization of IAA@Fe(III) NPs

SEM: A small amount of freeze-dried IAA@Fe(III) NPs was evenly coated on conductive tape, blown with high-pressure nitrogen, sputter-coated with gold, and observed under a HITACHI S-4800 SEM(Hitachi, Tokyo, Japan) [80].

FT-IR: Freeze-dried IAA@Fe(III) NPs and IAA raw material were analyzed by FT-IR using the KBr pellet method (4000–400 cm^−1^, 32 scans) to compare surface functional group changes.

XPS: Samples were analyzed using a Thermo Scientific ESCALAB 250Xi X-ray photoelectron spectrometer (Thermo Fisher Scientific, Waltham, MA, USA) with survey and high-resolution scans for C, O, and Fe. Data were charge-corrected and peak-fitted with Avantage software (version 5.9931, Thermo Fisher Scientific). 

XRD: Freeze-dried samples were recorded on a Bruker D8 ADVANCE diffractometer (Bruker, Karlsruhe, Germany) with Co target (λ = 1.79026 Å, 40 kV, 40 mA) for phase analysis.

DLS and Zeta potential: 2–5 mg of freeze-dried sample was dispersed in 2 mL deionized water (final concentration 1–2 mg/mL), sonicated for 5 min, and measured for hydrodynamic size and PDI by DLS at 25 °C. The same sample was transferred to a cuvette for zeta potential measurement.

UV-Vis: Using deionized water as blank, IAA@Fe(III) NPs dispersion (1–2 mg/mL), and IAA reference solution (1–2 mg/mL) were placed in quartz cuvettes and recorded for absorption spectra from 200 to 800 nm on a UV-2800A spectrophotometer (UNICO, Shanghai, China). [81].

#### 4.2.3. Performance Characterization of IAA@Fe(III) NPs

Encapsulation efficiency (EE%) and drug loading (DL%): The reaction system after standing at 4 °C was centrifuged at 4500 rpm for 20 min. An aliquot of 3 mL of the supernatant was precisely withdrawn, and its absorbance was measured at 375 nm. The concentration of free IAA was calculated by substituting the absorbance into the IAA standard curve (y = 1.4271x − 0.031, R^2^ = 0.9980, concentration range 0.5–20 μg/mL), and the amount of free IAA in the supernatant (W_1_) was subsequently calculated. Here, W_0_ is the initial amount of IAA added (15 mg), and W_2_ is the total mass of the lyophilized nanoparticles. The following formulas were used for calculation:

EE% = (W_0_ − W_1_)/W_0_ × 100%, DL% = (W_0_ − W_1_)/W_2_ × 100%,

where W_0_ is total IAA added, W_1_ is free IAA in supernatant, and W_2_ is the mass of freeze-dried nanoparticles.

Study on the sustained-release performance of nanoparticles: The in vitro release behavior of IAA@Fe(III) NPs was investigated using a dialysis method. Lyophilized nanoparticles (5 mg) were accurately weighed and dispersed in 10 mL of pH 6.8 PBS (0.01 M), then transferred into a dialysis bag (molecular weight cutoff: 3500 Da). After sealing both ends, the dialysis bag was placed in a 50 mL centrifuge tube containing 30 mL of the same pH buffer, and incubated at 37 °C with constant shaking at 150 rpm. At predetermined time points (every 10 min during the first hour, and then every 1 h thereafter), 3 mL aliquots were sampled, and the absorbance at 375 nm was measured. An equal volume of fresh PBS was immediately added to maintain a constant volume. The cumulative release rate at each time point was calculated by substituting the absorbance values into the standard curve.

### 4.3. Preparation and Characterization of Hydrogels

#### 4.3.1. Preparation of Hydrogels

Blank and drug-loaded composite hydrogels were prepared by thermogelation using Amy and CMCS as the matrix and borax as the crosslinker. Three drug-loading concentrations (0.25, 0.50, 0.75 mg/mL) were set. Corresponding amounts of freeze-dried IAA@Fe(III) NPs were dispersed in 10 mL deionized water by ultrasonication (240 W, 6 h) to obtain a nanoparticle dispersion. Borax (0.5 g) was added to the dispersion with stirring until completely dissolved and clear. CMCS (0.5 g) was then added with stirring to form a transparent solution. After cooling to room temperature, Amy (1 g) was added and dispersed uniformly. The mixture was placed on a 65 °C magnetic stirrer and reacted at 300 rpm for 30 min (post-heating method to avoid direct gelatinization of starch). After reaction, the mixture was cooled to room temperature to obtain the drug-loaded hydrogel. The blank hydrogel was prepared identically, except that the nanoparticle dispersion was replaced with an equal volume of deionized water. The hydrogels were sealed and stored at 4 °C for later use.

#### 4.3.2. Structural Characterization of Hydrogels

SEM: Freeze-dried hydrogel samples were fixed on sample stages with conductive tape, blown with a rubber bulb to remove loose powder, sputter-coated with gold, and observed for cross-sectional morphology under a Hitachi S-4800 SEM (Hitachi, Tokyo, Japan) [82].

FT-IR: Samples were analyzed by FT-IR using the KBr pellet method. Spectra were recorded from 4000 to 400 cm^−1^ (32 scans). Functional group features of amylopectin, CMCS, borax physical mixture, and the two hydrogels were compared.

XPS: Freeze-dried hydrogel samples were analyzed using a Thermo Scientific ESCALAB 250Xi X-ray photoelectron spectrometer (Thermo Fisher Scientific, Waltham, MA, USA) for survey spectra and high-resolution element scans (AC hydrogel: C, N, O, B; Amy/CMCS@NPs: C, N, O, B, Fe). Data were charge-corrected and peak-fitted with Avantage software (version 5.9931, Thermo Fisher Scientific, Waltham, MA, USA). 

#### 4.3.3. Characterization of Hydrogel Properties

Evaluation of swelling behavior: A gravimetric method was employed. The hydrogels (AC hydrogel and Amy/CMCS@NPs) were dried at 60 °C to constant weight, and the weight of the dried gels (W_0_, 2.0 g) was recorded. They were then immersed in 80 mL of PBS (pH 7.4) at 37 °C. At predetermined time points (every 10 min during the first hour, and then every 1 h thereafter), the hydrogels were removed, and the surface water was blotted dry with filter paper, after which the weight (W_t_) was recorded until constant weight was reached. The swelling ratio (SR) was calculated according to the following formula [83]:

SR = (W_t_ − W_0_)/W_t_ × 100%

Swelling curves were fitted with pseudo-first-order, pseudo-second-order, Fickian diffusion, and Higuchi models.

Rheological tests: Dynamic rheological characterization of AC hydrogel and Amy/CMCS@NPs was performed using a rotational rheometer (MCR 92, Anton Paar, Graz, Austria) (plate diameter 25 mm, gap 1.0 mm) at 25 °C. Time sweeps (0–200 s) measured storage modulus (G′), and loss modulus (G″) over time. Shear rate sweeps (0.1–100 s⁻¹) measured apparent viscosity. Frequency sweeps (ω = 0.1–100 rad/s, γ = 1%) measured G′ and G″ as functions of angular frequency. Strain sweeps (γ = 0.01–100%, ω = 10 rad/s) determined the linear viscoelastic region. 

Compression tests: Compression properties of AC hydrogel and Amy/CMCS@NPs were measured using a universal testing machine (WDT-10, Beijing Hongtai Shunda Technology Co., Ltd., Beijing, China). Cylindrical samples (diameter 15 mm, height 10 mm) were compressed at 5 mm/min to 50% strain (or until failure). Stress–strain curves were recorded, and compression modulus was calculated from the linear region (0–20% strain). 

Thermogravimetric analysis: Thermal stability of AC hydrogel and Amy/CMCS@NPs was evaluated using a Mettler TG-DSC 3+ simultaneous thermal analyzer (Mettler Toledo, Greifensee, Switzerland). Freeze-dried samples were placed in alumina crucibles and heated from 30 to 600 °C at 10 °C/min under N₂. TGA, DTG, and DSC curves were recorded to analyze thermal decomposition behavior and transition temperatures. 

#### 4.3.4. Functional Characterization of Hydrogels

Adhesion performance of the hydrogels was evaluated by two methods. Qualitative test: AC hydrogel and Amy/CMCS@NPs were adhered to various material surfaces and finger joint skin. Detachment under gravity and skin adhesion during joint bending were observed. Quantitative test: A porcine skin lap-shear model was used. Skin pieces (60 mm × 15 mm) were fixed onto glass plates. Hydrogel pieces (30 mm × 15 mm) were sandwiched between two skin pieces. Shear testing was performed at 2 mm/min with a 50 N force.

Self-healing performance was evaluated by macroscopic splitting-healing tests. Complete hydrogel pieces were cut into two sections. The cut surfaces were brought into close contact and left at room temperature for a set time. Interface fusion and mechanical integrity of the healed gel were observed.

Drug release testing: The in vitro release behavior of IAA from the Amy/CMCS@NPs hydrogel was investigated using a dialysis method. Drug-loaded hydrogel (2 g) was accurately weighed and immersed in a dialysis bag (molecular weight cutoff: 3500 Da) containing 10 mL of PBS (pH 7.4). After sealing, the dialysis bag was placed in a 50 mL centrifuge tube containing 30 mL of PBS, and incubated at 37 °C with constant shaking at 100 rpm. At predetermined time points (every 10 min during the first hour, and then every 1 h thereafter), 3 mL aliquots were sampled, and the absorbance at 375 nm was measured. An equal volume of fresh PBS was immediately added to replace the sampled volume. The cumulative release rate was calculated by substituting the absorbance values into the standard curve.

### 4.4. Study on Antibacterial Properties and Promotion of Wound Healing in Mice by Hydrogel

#### 4.4.1. The Antibacterial Properties of Hydrogels

The in vitro antibacterial activity of AC hydrogel and Amy/CMCS@NPs was evaluated using Gram-negative *E. coli* and Gram-positive *S. aureus* as model strains. All materials and media were sterilized by autoclaving (121 °C, 20 min).

MIC was determined by the agar dilution method. Different amounts of freeze-dried hydrogel samples were added to sterilized solid medium, sterilized at 115 °C for 30 min, mixed, poured into six-well plates, and allowed to solidify. Bacterial suspension (80 μL, ~10^6^–10^7^ CFU/mL) in logarithmic phase was spread evenly on the plate surface and incubated at 37 °C for 12–24 h. Inhibition zone diameters or colony growth were observed and recorded.

Antibacterial rate was determined as follows. Freeze-dried hydrogel (150 mg) was placed in a sterile container with 4 mL PBS (pH 7.4) and 1 mL bacterial suspension (~10^6^ CFU/mL), and incubated at 37 °C with shaking at 100 rpm for 12–24 h. An aliquot was spread on agar plates and incubated for another 12–24 h, followed by colony counting.

Antibacterial rate (%) = (1 − colony count of experimental group/colony count of blank control group) × 100%.

#### 4.4.2. Biocompatibility Evaluation of Hydrogels

Cell culture: L929 cells were routinely cultured in DMEM high-glucose medium with 10% FBS and 1% antibiotics at 37 °C in 5% CO_2_. Cells in logarithmic phase were used for subsequent experiments.

Cell viability was evaluated by MTT assay [84]. L929 cells (5 × 10^3^ per well) were seeded in 96-well plates and cultured for 24 h. The medium was replaced with 200 μL of hydrogel extracts at various concentrations. Fresh medium served as negative control, and medium without cells served as blank. Three replicate wells were used per group. After 24 h, 20 μL MTT (5 mg/mL) was added to each well and incubated for 4 h. The supernatant was discarded, and 150 μL DMSO was added to dissolve formazan crystals. Absorbance was measured at 570 nm. Cell viability was calculated as:

Cell viability (%) = (OD_sample_ − OD_blank_)/(OD_control_ − OD_blank_) × 100%

where OD_sample_, OD_blank_, and OD_control_ represent the absorbance values of the sample group, blank group, and control group, respectively.

Cell scratch assay: Cell migration was evaluated by scratch assay [85]. L929 cells were cultured in six-well plates to 90% confluence. A uniform scratch was made with a 200 μL sterile pipette tip. Cells were gently washed with PBS three times to remove detached cells. Experimental groups received complete medium with 3 mg/mL hydrogel extract, and controls received fresh medium. Three replicate wells were used per group. Scratch closure was photographed at 0, 2, 6, 12, and 24 h under an inverted optical microscope (×5 objective).

#### 4.4.3. Evaluation of Wound Repair Area in Mice

Full-thickness skin defect model: SPF-grade male ICR mice (6 weeks, 30 g) were used. After anesthesia, the dorsal hair was shaved and the skin was disinfected with iodine. An 8 mm diameter skin punch was used to create full-thickness skin defects down to the subcutaneous fascia layer.

Wound healing rate: Mice were divided into five groups. Control group: wounds rinsed daily with saline. Positive control: commercial cationic hydrogel. Experimental groups: Amy/CMCS hydrogel, Amy/CMCS@NPs hydrogel, and Amy/CMCS/IAA hydrogel applied under sterile conditions. Dressings were changed daily. Wounds were photographed, and wound areas were measured with Fiji distribution of ImageJ (version 20260114-1317, National Institutes of Health, Bethesda, MD, USA).

Healing rate = (A_0_ − A_t_)/A_0_ × 100%

#### 4.4.4. Histological Evaluation by H and E Staining and Masson Staining

Mouse wound tissue sectioning: Skin tissues around the wound were collected on days 3 and 7, fixed in 4% paraformaldehyde (PFA), dehydrated, embedded, and sectioned. Samples were stained with H and E to evaluate epidermal regeneration and wound re-epithelialization, and with Masson’s trichrome to quantify collagen deposition area. Sections were observed and photographed under a microscope, and pathological changes were recorded and analyzed using ImageJ software.

#### 4.4.5. Mouse Wound Immunohistochemical Test

At designated time points (days 3 and 7), wound tissues were collected, fixed in 4% PFA, dehydrated, embedded, and sectioned. Immunohistochemical staining for IL-1β and TNF-α was performed to evaluate wound inflammation. Sections were observed and photographed under a microscope for histopathological analysis.

#### 4.4.6. Immunofluorescence Staining

On days 3 and 7, mice were euthanized by cervical dislocation. Skin tissues around the wound were collected, fixed in 4% PFA, dehydrated, embedded, sectioned, and stained by immunofluorescence. Samples were stained with CD31 and VEGF. Images were acquired under a fluorescence microscope and analyzed using ImageJ software.

### 4.5. Statistic Analysis

All experiments were independently repeated at least three times. Data were expressed as mean ± SD. One-way ANOVA was performed using IBM SPSS 18.0, followed by Tukey’s post-hoc test for multiple comparisons. Significance was set at *p* < 0.05 (statistically significant) and *p* < 0.01 or *p* < 0.001 (highly significant). Graphs were generated using Origin 2021.

## Figures and Tables

**Table 1:** Atomic percentage contents of C, O, and Fe in nanoparticles.

Name	Atomic Concentration (%)
C	67.77
O	31.51
Fe	0.73

**Table 2:** Atomic percentage contents of C, N, O, and B elements in AC hydrogel.

Name	Atomic Concentration (%)
C	53.28%
N	6.75%
O	35.54%
B	4.43%

**Table 3:** Atomic percentage contents of C, N, O, B, and Fe in Amy/CMCS@NPs.

Name	Atomic Concentration (%)
C	54.97
N	5.24
O	38.84
B	0.80
Fe	0.15

**Table 4:** The fitting parameters of the kinetic model for AC hydrogel.

Kinetic Model	Parameters	Value
Pseudo-first-order	K1	0.02
	R2	0.98
Pseudo-second-order	Me	411.52
	K2	0.54 × 10^−4^
	R2	0.99
Korsmeyer–Peppas	*n*	0.54
	KKP	0.06
	R2	0.98
Higuchi	KH	0.07
	R2	0.98

**Table 5:** The fitting parameters of the kinetic model for Amy/CMCS@NPs hydrogel.

Kinetic Model	Parameters	Value
Pseudo-first-order	K1	0.06
	R2	0.98
Pseudo-second-order	Me	411.52
	K2	0.33 × 10^−4^
	R2	0.99
Korsmeyer–Peppas	*n*	0.54
	KKP	0.03
	R2	0.96
Higuchi	KH	0.04
	R2	0.95

## Data Availability

The raw data supporting the conclusions of this article will be made available by the authors on request.

## Supplementary Materials

The following supporting information can be downloaded at: https://www.mdpi.com/article/10.3390/gels12070637/s1, Figure S1: Chemical structure of IAA; Figure S2. XPS Survey Spectrum of IAA@Fe(III) NPs; Figure S3. Full wavelength scanning spectra of IAA and nanoparticles; Figure S4. Preparation of hydrogels (a) AC hydrogel; (b) Amy/CMCS@NPs; Figure S5. XPS survey spectrum of AC hydrogel; Figure S6. XPS Survey Spectrum of Amy/CMCS@NPs composite hydrogel; Figure S7. Swelling curves of AC hydrogel; Figure S8. Swelling Curve of Amy/CMCS@NPs composite hydrogel; Figure S9. The fitting curves of different swelling kinetics models for AC hydrogel: (a) pseudo-first-order swelling kinetics, (b) pseudo-second-order swelling kinetics, (c) solution diffusion model, (d) Higuchi model; Figure S10. Swelling Kinetic Fitting Curves of Amy/CMCS@NPs hydrogel, (a) Pseudo-first-order Kinetic Model, (b) Pseudo-second-order Kinetic Model, (c) Intraparticle Diffusion Model, and d) Higuchi Model; Figure S11. Time sweep rheological analysis at 25 °C (a), shear rate-viscosity curve (b), frequency sweep rheological analysis (c) and amplitude sweep rheological curve (d) of AC hydrogel; Figure S12. Time sweep rheological analysis at 25 °C (a), shear rate-viscosity curve (b), frequency sweep rheological analysis (c) and amplitude sweep rheological curve (d) of Amy/CMCS@NPs hydrogel; Figure S13. Compressive stress-strain curves of AC hydrogel; Figure S14. Compressive Stress-Strain Curve of Amy/CMCS@NPs hydrogel; Figure S15. Thermal Analysis of AC hydrogel, (a) TGA, (b) DSC, and (c) DTG; Figure S16. Thermal Analysis of Amy/CMCS@NPs hydrogel, (a) TGA, (b) DSC, and (c) DTG; Figure S17. Adhesive properties of AC hydrogel (a) adhesion to skin; (b) adhesion to different substrates; Figure S18. Adhesion Performance of Amy/CMCS@NPs hydrogel to Different Materials; Figure S19. Self-healing performance of AC hydrogel; Figure S20. Self-healing performance of Amy/CMCS@NPs hydrogel; Figure S21. Drug Release Performance of Amy/CMCS@NPs hydrogel.
