# Supplementary material for: Preparation of Multifunctional Hydrogel Loaded with Isochlorogenic Acid A/Fe3+ Co-Assembled Nanoparticles and Its Application in Skin Wound Repair
Source: Gels. 2026 Jul 16;12(7):637. doi: 10.3390/gels12070637 (PMC13407546; doi:10.3390/gels12070637)
Supplement: Supplementary file 1 [file gels-12-00637-s001.zip › gels-4391165-supplementary.pdf]

Article

Preparation of multifunctional hydrogel loaded with Isochlorogenic acid A/Fe^{3+} co-assembled nanoparticles and its application in skin wound repair

Hui Li [1,†], Danli Peng [1†], Zhijia Wang [1], Yuping Zhang [1], Xingyu Yang [1], Yongmei Jiang [1], Xin Zhang [1], Lei Zhu [1], Yanlei Guo [3*], Yongai Xiong [1*] and Gang Wang [1, 2*]

[1] School of Pharmacy, Zunyi Medical University, Zunyi 563000, China;
[2] Chongqing Academy of Chinese Materia Medica, No.34 Nanshan Road, Nan'an District, Chongqing 400065, China
[3] Guizhou Key Laboratory of Modern Traditional Chinese Medicine Creation, Zunyi 563000, China

* Correspondence: guoyanlei1210@163.com (Y.G.); yaxiong@zmu.edu.cn (Y.X.); wg8855350@163.com (G.W.); Tel.: +86-23-89029031 (Y.G.); +86-851-28642515 (Y.X.); +86-136-7852-6088 (G.W.)
[†] These authors contributed equally to this work.

Contents

Figures

Figure S1. Chemical structure of IAA

Figure S2. XPS Survey Spectrum of IAA@Fe(III) NPs

Figure S3. Full wavelength scanning spectra of IAA and nanoparticles

Figure S4. Preparation of hydrogels (a) AC hydrogel; (b) Amy/CMCS@NPs.

Figure S5. XPS survey spectrum of AC hydrogel

Figure S6. XPS Survey Spectrum of Amy/CMCS@NPs composite hydrogel

Figure S7. Swelling curves of AC hydrogel

Figure S8. Swelling Curve of Amy/CMCS@NPs composite hydrogel

Figure S9. The fitting curves of different swelling kinetics models for AC hydrogel: (a) pseudo-first-order swelling kinetics, (b) pseudo-second-order swelling kinetics, (c) solution diffusion model, (d) Higuchi model

Figure S10. Swelling Kinetic Fitting Curves of Amy/CMCS@NPs hydrogel, (a) Pseudo-first-order Kinetic Model, (b) Pseudo-second-order Kinetic Model, (c) Intraparticle Diffusion Model, and d) Higuchi Model

Figure S11. Time sweep rheological analysis at 25 °C (a), shear rate-viscosity curve (b), frequency sweep

rheological analysis (c) and amplitude sweep rheological curve (d) of AC hydrogel

Figure S12. Time sweep rheological analysis at 25 ° C (a), shear rate-viscosity curve (b), frequency sweep rheological analysis (c) and amplitude sweep rheological curve (d) of Amy/CMCS@NPs hydrogel

Figure S13. Compressive stress-strain curves of AC hydrogel

Figure S14. Compressive Stress-Strain Curve of Amy/CMCS@NPs hydrogel

FigureS15. Thermal Analysis of AC hydrogel, (a) TGA, (b) DSC, and (c) DTG

FigureS16. Thermal Analysis of Amy/CMCS@NPs hydrogel, (a) TGA, (b) DSC, and (c) DTG

Figure S17. Adhesive properties of AC hydrogel (a) adhesion to skin; (b) adhesion to different substrates

FigureS18. Adhesion Performance of Amy/CMCS@NPs hydrogel to Different Materials

Figure S19. Self-healing performance of AC hydrogel

Figure S20. Self-healing performance of Amy/CMCS@NPs hydrogel

Figure S21. Drug Release Performance of Amy/CMCS@NPs hydrogel

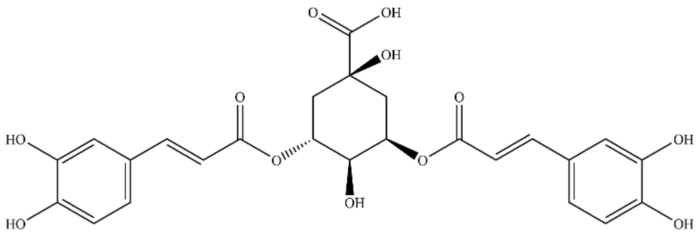

Figure S1. Chemical structure of IAA

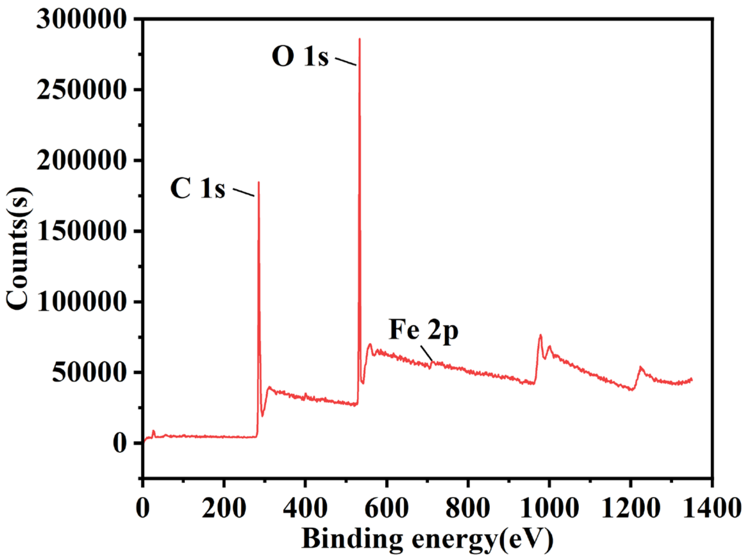

Figure S2. XPS Survey Spectrum of IAA@Fe(III) NPs

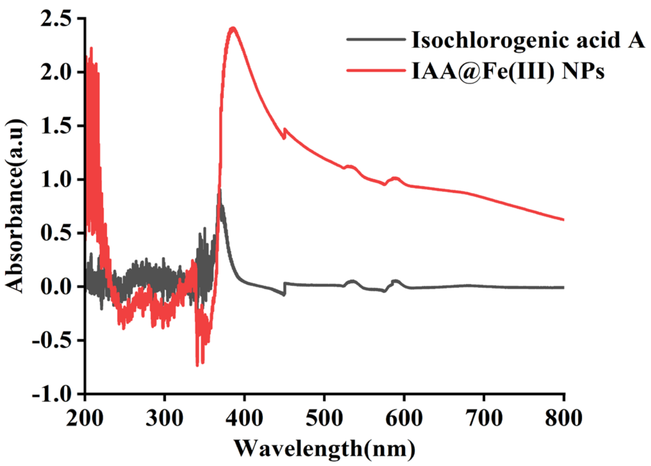

Figure S3. Full wavelength scanning spectra of IAA and nanoparticles

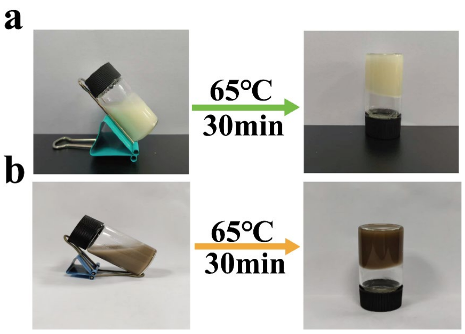

Figure S4. Preparation of hydrogels (a) AC hydrogel; (b) Amy/CMCS@NPs.

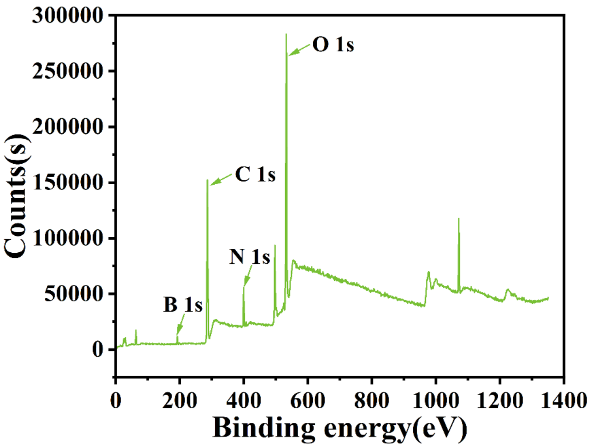

Figure S5. XPS survey spectrum of AC hydrogel

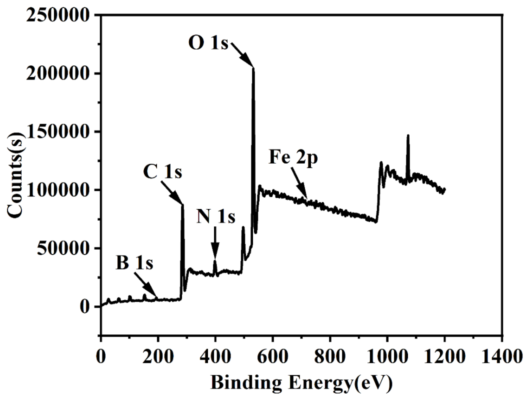

Figure S6. XPS Survey Spectrum of Amy/CMCS@NPs composite hydrogel

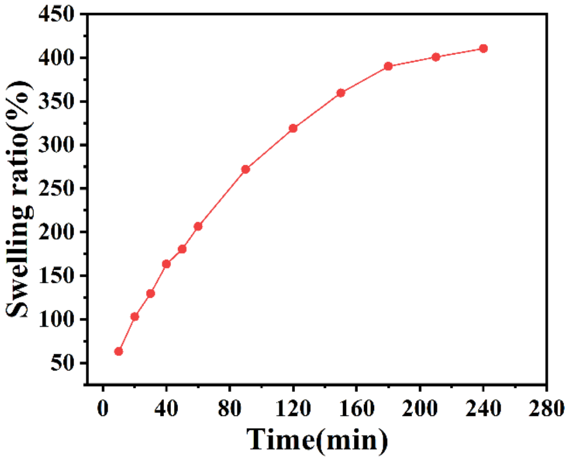

Figure S7. Swelling curves of AC hydrogel

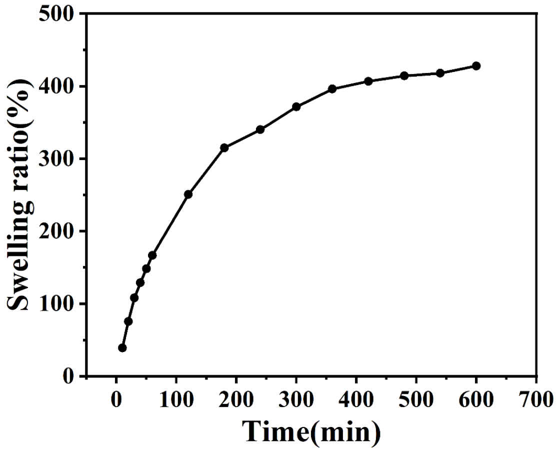

Figure S8. Swelling Curve of Amy/CMCS@NPs composite hydrogel

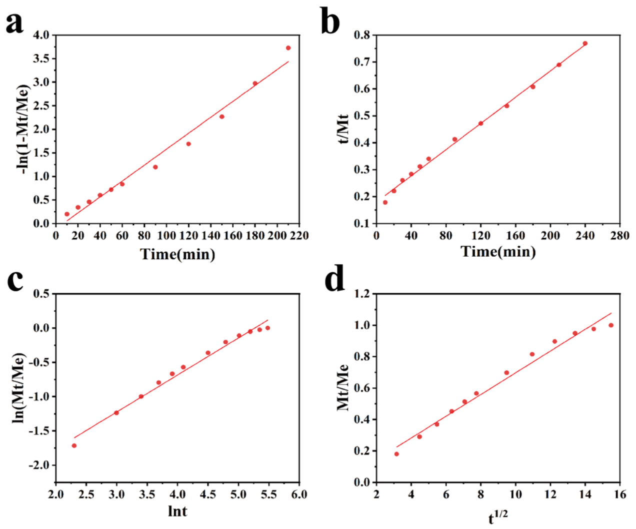

Figure S9. The fitting curves of different swelling kinetics models for AC hydrogel: (a) pseudo-first-order swelling kinetics, (b) pseudo-second-order swelling kinetics, (c) solution diffusion model, (d) Higuchi model

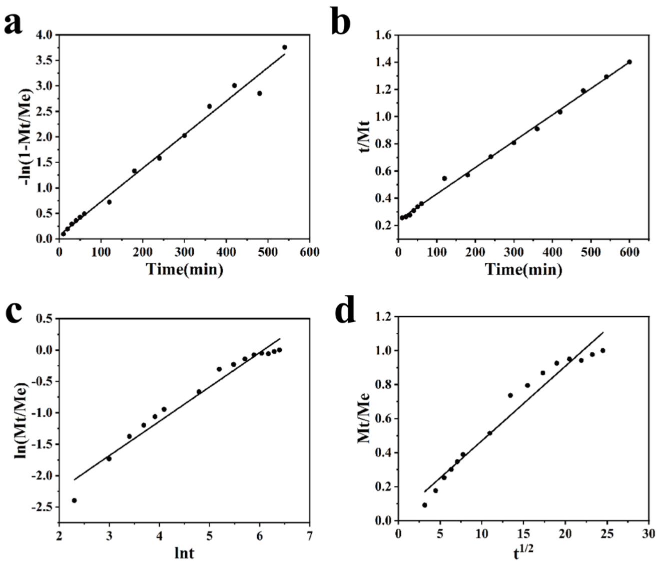

Figure S10. Swelling Kinetic Fitting Curves of Amy/CMCS@NPs hydrogel, (a) Pseudo-first-order Kinetic Model, (b) Pseudo-second-order Kinetic Model, (c) Intraparticle Diffusion Model, and d) Higuchi Model

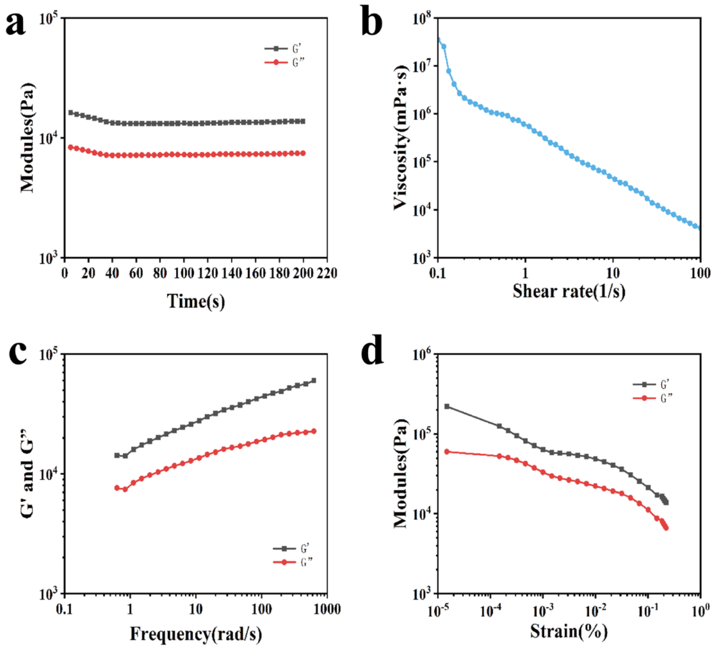

Figure S11. Time sweep rheological analysis at 25 °C (a), shear rate-viscosity curve (b), frequency sweep rheological analysis (c) and amplitude sweep rheological curve (d) of AC hydrogel

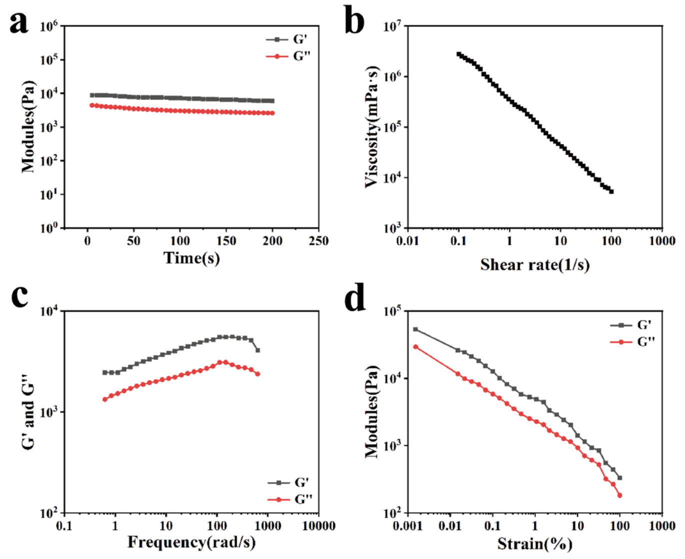

Figure S12. Time sweep rheological analysis at 25 ° C (a), shear rate-viscosity curve (b), frequency sweep rheological analysis (c) and amplitude sweep rheological curve (d) of Amy/CMCS@NPs hydrogel

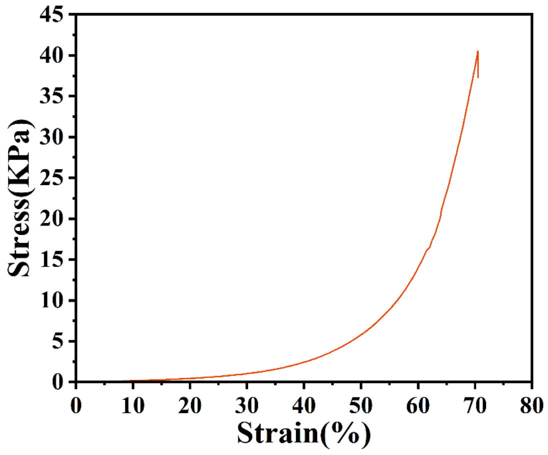

Figure S13. Compressive stress-strain curves of AC hydrogel

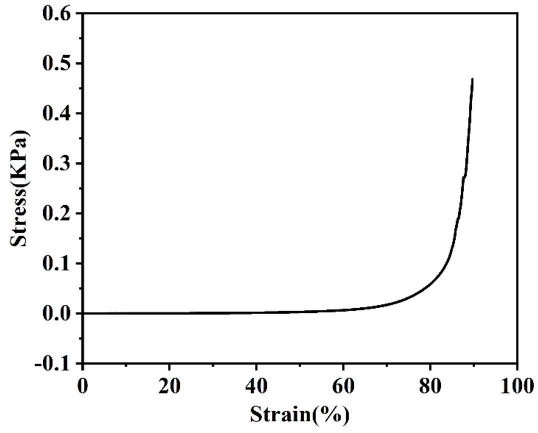

Figure S14. Compressive Stress-Strain Curve of Amy/CMCS@NPs hydrogel

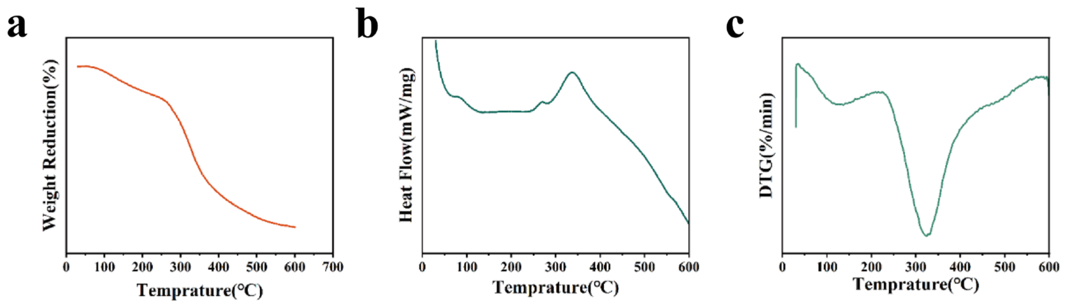

FigureS15. Thermal Analysis of AC hydrogel, (a) TGA, (b) DSC, and (c) DTG

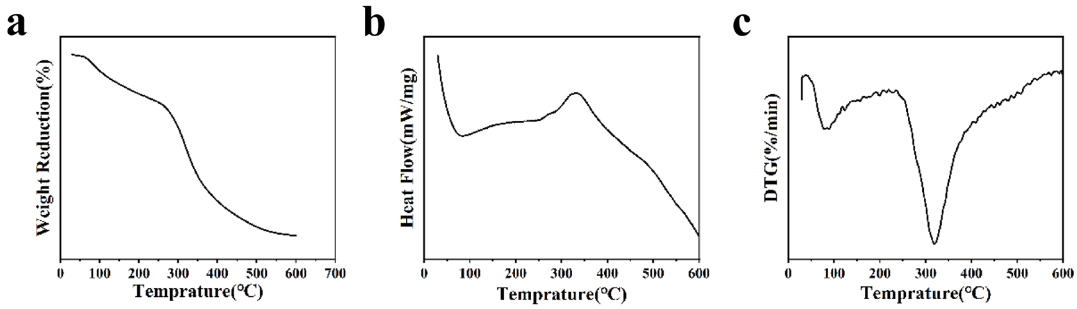

FigureS16. Thermal Analysis of Amy/CMCS@NPs hydrogel, (a) TGA, (b) DSC, and (c) DTG

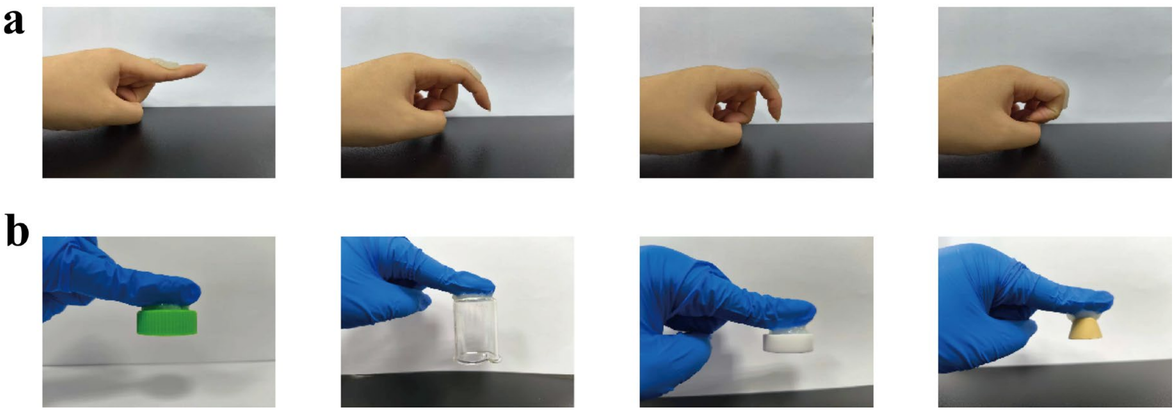

Figure S17. Adhesive properties of AC hydrogel (a) adhesion to skin; (b) adhesion to different substrates

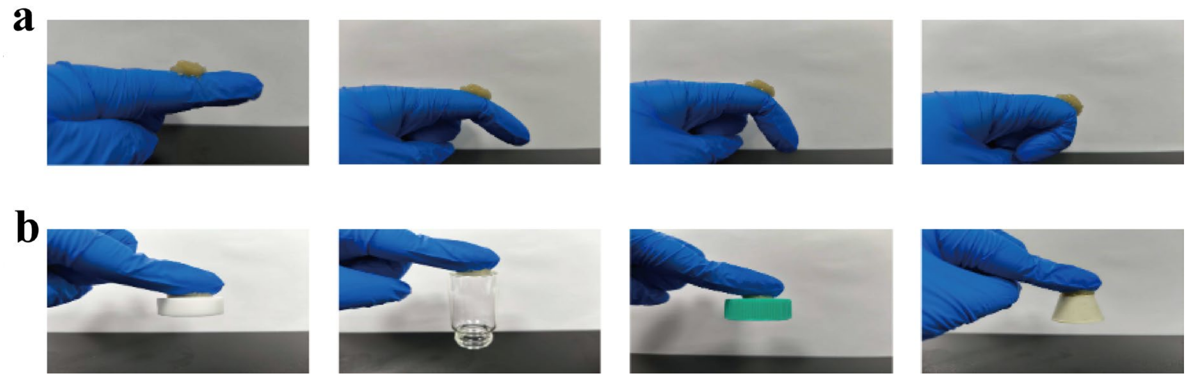

FigureS18. Adhesion Performance of Amy/CMCS@NPs hydrogel to Different Materials

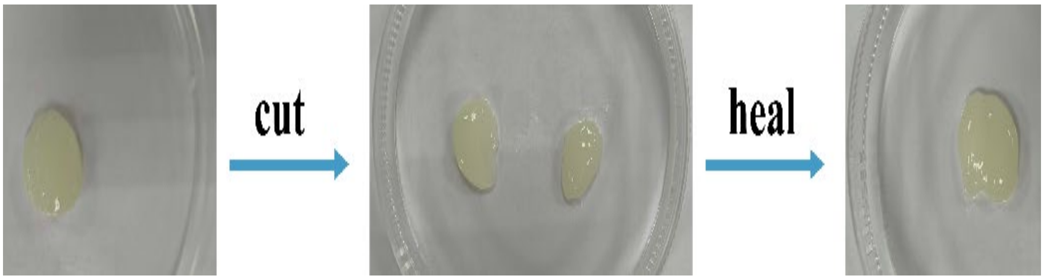

Figure S19. Self-healing performance of AC hydrogel

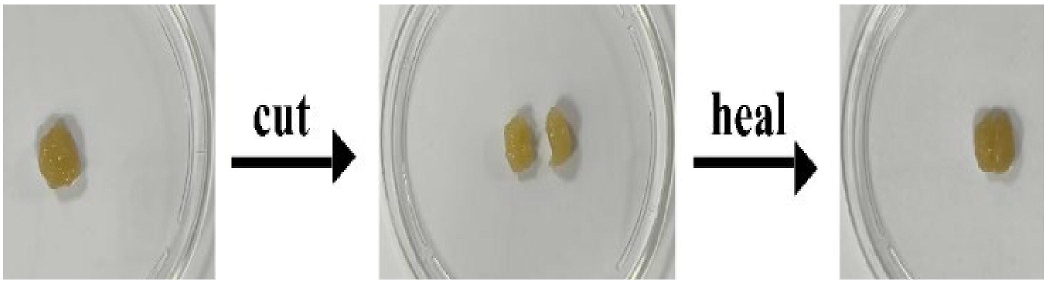

Figure S20. Self-healing performance of Amy/CMCS@NPs hydrogel

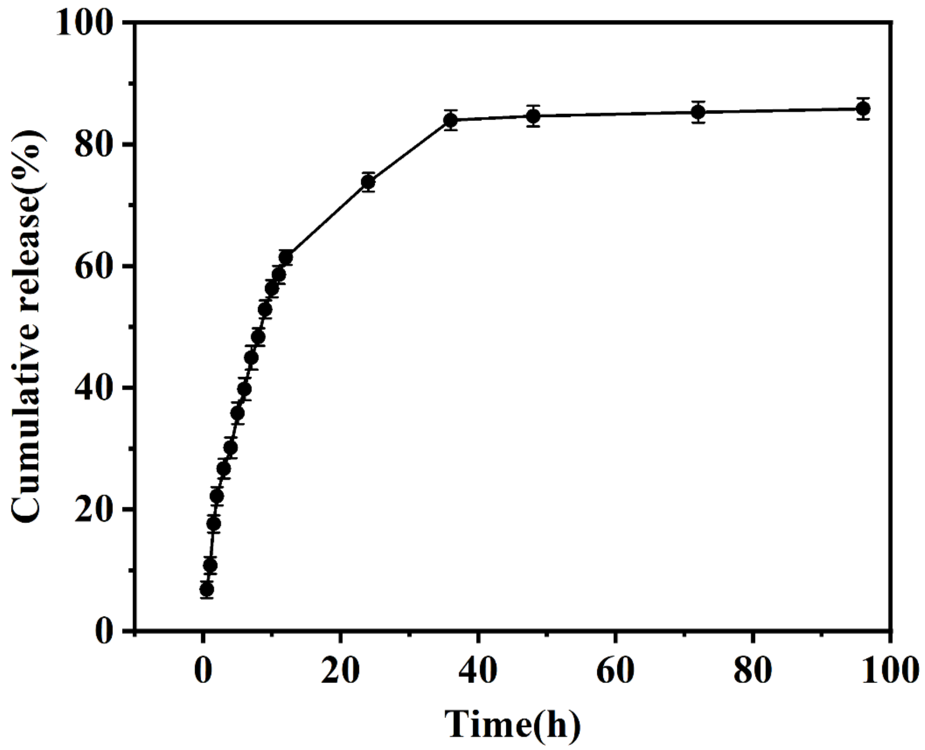

Figure S21. Drug Release Performance of Amy/CMCS@NPs hydrogel
